# Suppressive effects of the obese tumor microenvironment on CD8 T cell infiltration and effector function

**DOI:** 10.1084/jem.20210042

**Published:** 2022-02-01

**Authors:** Lydia Dyck, Hannah Prendeville, Mathilde Raverdeau, Mieszko M. Wilk, Roisin M. Loftus, Aaron Douglas, Janet McCormack, Bruce Moran, Michael Wilkinson, Evanna L. Mills, Michael Doughty, Aurelie Fabre, Helen Heneghan, Carel LeRoux, Andrew Hogan, Edward T. Chouchani, Donal O’Shea, Donal Brennan, Lydia Lynch

**Affiliations:** 1 School of Biochemistry and Immunology, Trinity College Dublin, Dublin, Ireland; 2 Research Pathology Core Facility, Conway Institute, University College Dublin, Dublin, Ireland; 3 Department of Pathology, St. Vincent’s University Hospital, Dublin, Ireland; 4 University College Dublin Gynaecological Oncology Group, University College Dublin School of Medicine, Mater Misericordiae University Hospital, Dublin, Ireland; 5 Department of Cancer Biology, Dana-Farber Cancer Institute, Boston, MA; 6 Department of Cell Biology, Harvard Medical School, Boston, MA; 7 Department of Cellular Pathology, Mater Misericordiae University Hospital, Dublin, Ireland; 8 School of Medicine, St. Vincent's University Hospital and University College Dublin, Dublin, Ireland; 9 Human Health Institute, Department of Biology, Maynooth University, Maynooth, Ireland; 10 National Children’s Research Centre, Dublin, Ireland; 11 Systems Biology Ireland, School of Medicine, University College Dublin, Dublin, Ireland; 12 Brigham and Women’s Hospital, Boston, MA; 13 Harvard Medical School, Boston, MA

## Abstract

Obesity is one of the leading preventable causes of cancer; however, little is known about the effects of obesity on anti-tumor immunity. Here, we investigated the effects of obesity on CD8 T cells in mouse models and patients with endometrial cancer. Our findings revealed that CD8 T cell infiltration is suppressed in obesity, which was associated with a decrease in chemokine production. Tumor-resident CD8 T cells were also functionally suppressed in obese mice, which was associated with a suppression of amino acid metabolism. Similarly, we found that a high BMI negatively correlated with CD8 infiltration in human endometrial cancer and that weight loss was associated with a complete pathological response in six of nine patients. Moreover, immunotherapy using anti–PD-1 led to tumor rejection in lean and obese mice and partially restored CD8 metabolism and anti-tumor immunity. These findings highlight the suppressive effects of obesity on CD8 T cell anti-tumor immunity, which can partially be reversed by weight loss and/or immunotherapy.

## Teaser

Dyck et al. describe suppressive effects of obesity on CD8 T cells in mouse cancer models and human endometrial cancer. Immunotherapy using anti–PD-1 is effective in obesity and partially restores CD8 T cell function and metabolism in mice.

## Introduction

Overweight and obesity are a major health burden affecting ∼40% of the adult population worldwide (World Health Organization, 2016). Consequently, there is an increase in obesity-associated diseases, including not only type 2 diabetes, cardiovascular disease, and autoimmunity, but also cancer. Obesity is associated with an increased cancer risk, most significantly for endometrial, esophageal, and colorectal cancers (Calle and Kaaks, 2004; De Pergola and Silvestris, 2013). Multiple links between obesity and cancer risk have been identified, including increased levels of growth hormones, leptin, insulin, and adipokines, which can directly contribute to tumor transformation and progression (Dyck and Lynch, 2018; Font-Burgada et al., 2016). At the same time, recent studies suggest that obesity might also indirectly contribute to tumorigenesis by suppressing anti-tumor immunity. We have recently demonstrated that obesity impairs anti-tumor responses by natural killer (NK) cells by induction of peroxisome proliferator-activated receptor–driven lipid metabolism leading to metabolic paralysis in NK cells (Michelet et al., 2018). Moreover, Wang et al. (2019) suggested that obesity promotes T cell exhaustion through leptin-induced up-regulation of PD-1. Thus, obesity may be a double hit for tumor promotion through accelerating tumor proliferation (Font-Burgada et al., 2016) and metastasis (Pascual et al., 2017) and impairing the anti-tumor immune response.

In established tumors, CD8 T cells are often found to be dysfunctional or “exhausted.” Exhausted T cells are characterized by the expression of multiple immune checkpoints, such as PD-1, LAG-3 and Tim-3, and a failure to proliferate and produce effector molecules, such as IFN-γ (Wherry and Kurachi, 2015). Cancer immunotherapy has revolutionized the treatment of certain cancers by reinvigorating adaptive immune responses against the tumor. Immune checkpoint blockade (ICB) using anti–PD-1 antibodies has induced objective responses and significantly prolonged survival rates in melanoma and non–small cell lung cancer patients (Garon et al., 2015; Herbst et al., 2016; Larkin et al., 2015; Robert et al., 2015a). Cancer immunotherapy and ICB are likely to form the backbone of therapies in multiple cancers; however, there is still a significant proportion of patients who do not respond to ICB-based therapies (Callahan et al., 2016). Thus, there is a need to identify factors that might affect responsiveness to immunotherapy. Given that obesity induced metabolic paralysis in NK cells, we hypothesized that CD8 T cell responses may be similarly affected. Because PD-1 ligation has been shown to impair T cell metabolic reprogramming and ICB may partially work through reinvigoration of metabolic pathways (Bengsch et al., 2016; Chang et al., 2015; Patsoukis et al., 2015), immune cells in obesity may not respond to ICB. Thus, the aim of this study was to investigate the effects of obesity on adaptive immune responses by CD8 T cells. Improved CD8 T cell responses have been associated with the responsiveness to ICB in mouse models and in the clinical setting (Duraiswamy et al., 2013; Fumet et al., 2018; Gubin et al., 2014; Woo et al., 2012). We found that in addition to NK cells, CD8 T cells were greatly diminished in tumors from obese mice. Residual tumor-infiltrating CD8 T cells were functionally and metabolically impaired in obesity and failed to control tumor growth. We investigated whether obesity-induced immunosuppression affected the responsiveness to cancer immunotherapy. Surprisingly, anti–PD-1 treatment led to complete tumor eradication and immune memory in both lean and obese mice. Anti–PD-1 restored CD8 T cell infiltration, proinflammatory cytokine production, and metabolic activity, and mice that previously cleared tumors were protected from rechallenge. In humans, endometrial cancer has the strongest association with obesity (De Pergola and Silvestris, 2013). Importantly, we also found an inverse correlation between CD8 T cell infiltration and body mass index (BMI) in endometrial tumors. In addition, we found that weight loss was associated with increased PD-L1 expression and infiltration of T cells in tumors. Thus, in humans and mice, obesity is associated with a “cold” tumor phenotype. However, our findings highlight that immune dysfunction in obesity is reversible. While obesity suppressed CD8 T cells in tumors and promoted tumor growth, immunotherapy was able to reverse these defects, resulting in tumor rejection. Similarly, weight loss in endometrial cancer led to tumor regression and restored immune cell infiltration.

## Results

### High-fat diet (HFD)–induced obesity increases tumor growth and decreases tumor-infiltrating lymphocytes (TILs)

**Figure 1.**
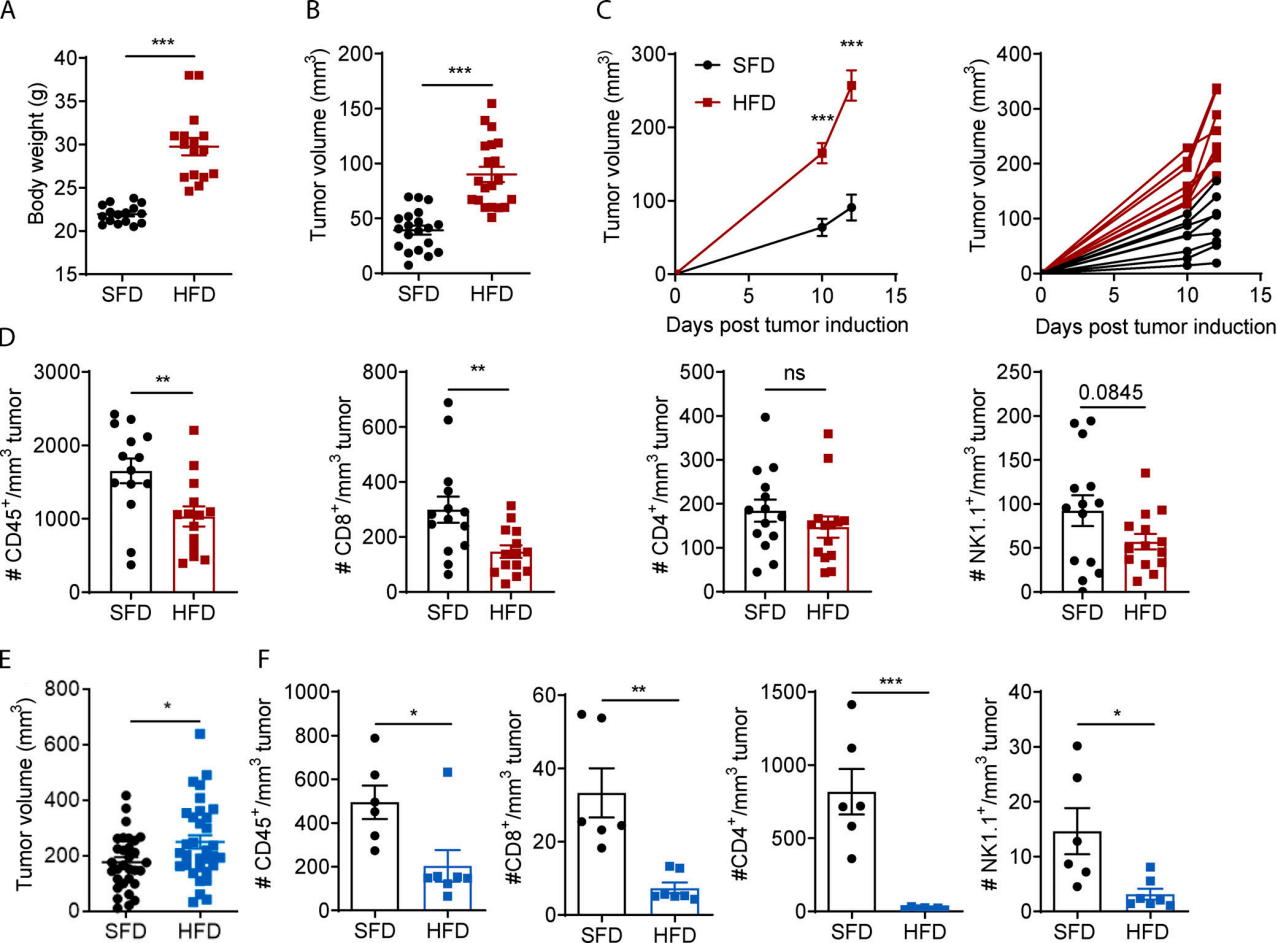
**HFD-induced obesity increases tumor growth and decreases immune cell infiltration in mice. (A–D)** C57BL/6 mice were fed an HFD (*n* = 14–16) or an SFD (*n* = 14–16) for 6–9 wk, and MC38 tumors were injected. Graphs depict weight at 9 wk for HFD (A), tumor volume on day 7 after tumor inoculation (B), and tumor growth progression (C). **(D)** Tumors from C were dissected, and immune cell infiltration was analyzed by flow cytometry. **(E)** C57BL/6 mice were fed an HFD or SFD for 10–13 wk, and mice were injected s.c. with B16-F10 tumor cells. Graph indicates tumor volume on day 11 after tumor inoculation from five pooled experiments (SFD, *n* = 31; HFD, *n* = 33). **(F)** Tumors from one experiment in E were dissected, and immune cell infiltration was analyzed by flow cytometry (SFD, *n* = 6; HFD, *n* = 7). Data are shown as individual mice (dots) and mean ± SEM. **(A, B, D, and E)** Unpaired Student’s *t* test. **(C)** Two-way ANOVA. *, P < 0.05; **, P < 0.01; ***, P < 0.001. This experiment was performed five times in the MC38 model and six times in the B16 model.

To study the effects of obesity on tumor progression and anti-tumor immunity, we fed mice an HFD to induce obesity and transplanted tumor cells once mice were obese. After 9 wk on an HFD, mice significantly increased in body weight (Fig. 1 A). Mice were injected with MC38 colon carcinoma cells, and, 7 d after tumor inoculation, the tumor volume was significantly greater and tumor growth progressed quicker in HFD-fed mice than in standard fat diet (SFD)–fed mice (Fig. 1, B and C). Similarly, B16-F10 tumors were significantly larger in obese than in lean mice 11 d after inoculation, although the increase in tumor growth was less severe than in MC38 tumors (Fig. 1 E). To assess whether HFD-induced obesity affected the immune response, immune infiltrates in MC38 and B16-F10 tumors were analyzed by flow cytometry. MC38 tumors in HFD-fed mice had a significantly reduced density of infiltrating total leukocytes (CD45^+^) and CD8 T cells and minor reductions in CD4 and NK cells (Fig. 1 D). Similarly, in B16-F10 tumors, the number of total infiltrating leukocytes, CD8 T cells, CD4 T cells, and NK cells was significantly reduced in HFD-fed mice compared with SFD-fed mice (Fig. 1 F). These findings show that obesity promotes tumor growth in multiple tumor models and suppresses immune cell infiltration and/or persistence of immune cells in the tumor.

**Figure 2.**
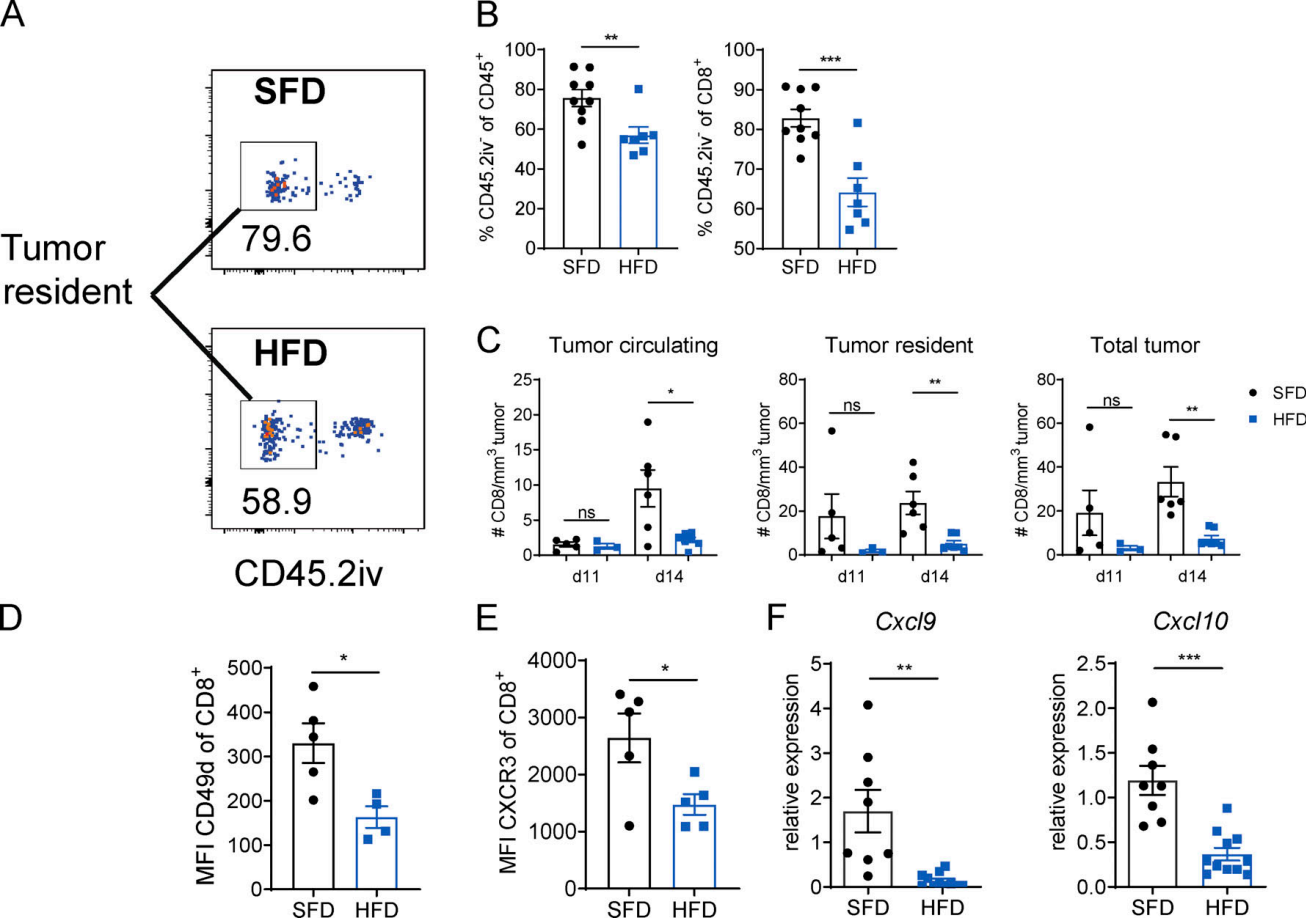
**HFD-induced obesity suppresses immune cell trafficking to the tumor.** C57BL/6 mice were fed an HFD or SFD for 9–12 wk, and B16-F10 tumors were injected. CD45.2-PE was injected i.v. 10 min before culling. This experiment was performed twice. **(A)** Exemplary image of flow cytometric analysis of tumors from SFD- and HFD-fed mice. Boxes indicate CD45.2iv^−^ tumor-resident population. **(B)** Proportion of tumor-resident leukocytes (CD45.2iv^−^) and CD8^+^ T cells in B16-F10 tumors. **(C)** Number of tumor-circulating, tumor-resident, and total CD8^+^ T cells in B16-F10 tumors. **(D and E)** Flow cytometric analysis of CXCR3 and CD49d expression on CD8^+^ T cells in B16.F10 tumors. **(F)** qPCR on total B16-F10 tumors from SFD-fed (*n* = 8) and HFD-fed (*n* = 11) mice from two pooled experiments. Data are shown as individual mice (dots) and mean ± SEM. Significance was calculated using an unpaired Student’s *t* test. *, P < 0.05; **, P < 0.01; ***, P < 0.001.

**Figure S1.**
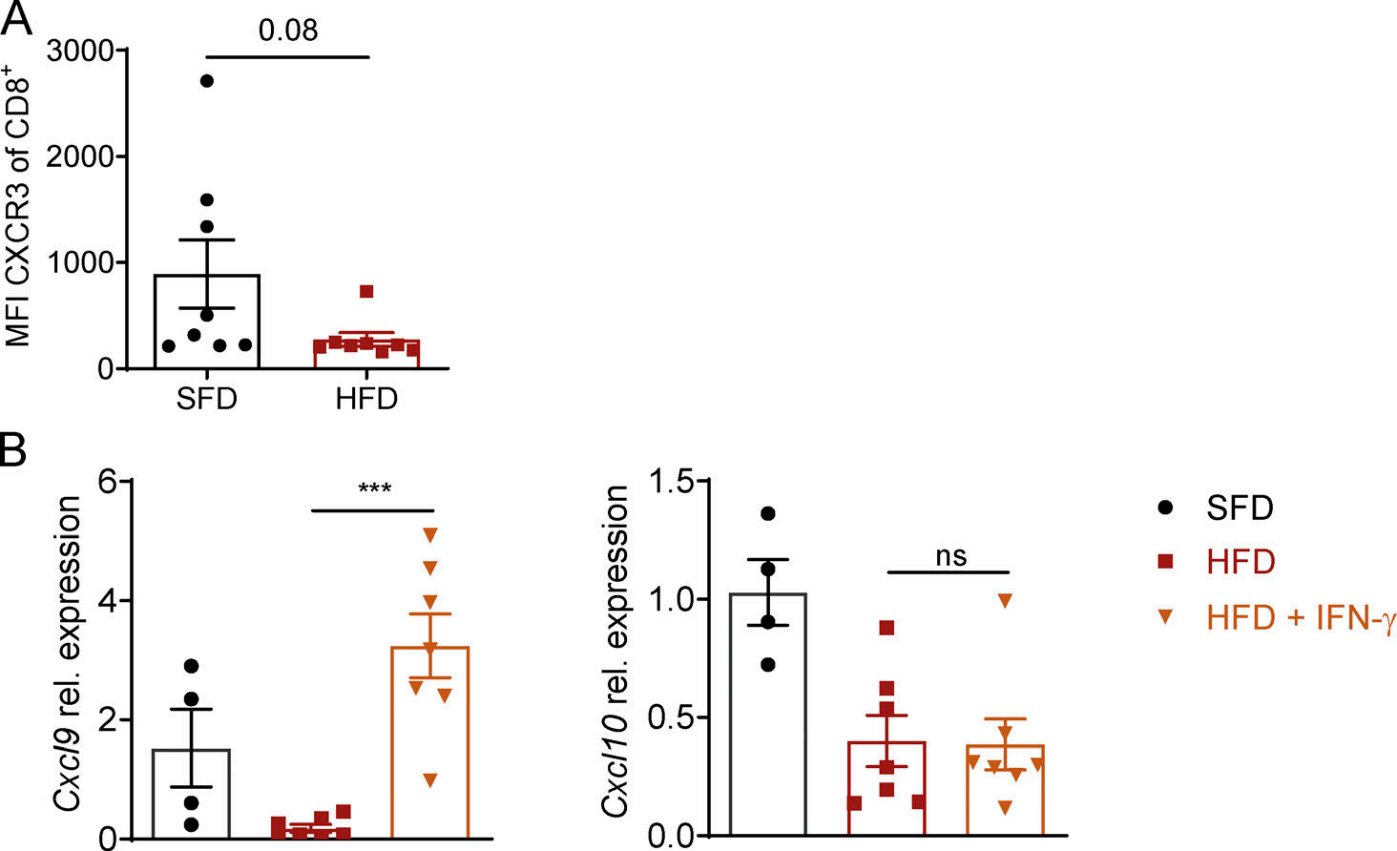
**Chemokine and chemokine receptor expression in tumors of HFD-fed mice.** **(A)** Flow cytometric analysis of CXCR3 expression by CD8^+^ T cells on day 17 after tumor injection in MC38 tumors of SFD-fed (*n* = 7) or HFD-fed (*n* = 8) mice. Dots represent individual mice. P = 0.08 using unpaired Student’s *t* test. **(B)** SFD and HFD mice were injected with B16-F10 tumors, and HFD mice were injected s.c. with rmIFN-γ on days 5, 8, 11, and 13. Experiment was performed twice (*n* = 4–7 per group). Relative expression of *Cxcl9* and *Cxcl10* from total tumors on day 14 after tumor injection. Data are shown as individual mice and mean ± SEM; one-way ANOVA. ***, P < 0.001.

To investigate whether a defect in extravasation contributed to the reduction in infiltrating leukocytes in obesity, we measured the proportion of tissue-resident immune cells within B16-F10 tumors. Mice were injected i.v. with a fluorescent PE-conjugated antibody against CD45.2 and sacrificed 10 min later. The i.v. injected antibodies stained all circulating immune cells but not tumor-resident immune cells, which were identified by gating on CD45.2iv^−^ cells (Fig. 2 A). We found that obese mice had a significantly lower percentage of tumor-resident leukocytes and CD8 T cells than lean mice (Fig. 2, A and B). Interestingly, on day 11 after tumor induction, there was a slight but not significant reduction in CD8 T cells in the total tumor and tumor tissue but no difference in the number of cells in the blood vessels in the tumor (Fig. 2 C). However, on day 14 after tumor induction, the number of CD8 T cells in the total tumor, tumor tissue, and tumor circulation was reduced in obese mice compared with lean mice (Fig. 2 C). These findings suggest that T cell extravasation was impaired in obesity as early as 11 d after tumor induction and that T cell recruitment to the tumor site was significantly suppressed by day 14 in obesity. To further elucidate whether obesity affected T cell trafficking, we measured the expression of the chemokine receptor CXCR3, which has been shown to mediate CD8 trafficking to tumors and the extravasation into the tumor tissue (Chamoto et al., 2017; Chheda et al., 2016; Mikucki et al., 2015), and the integrin CD49d on CD8 T cells, which is involved in T cell transmigration (Sökeland and Schumacher, 2019). CD8 T cells expressed lower levels of CXCR3 and CD49d in HFD than in SFD mice (Fig. 2, D and E; and Fig. S1 A). Moreover, there was significantly reduced expression of the CXCR3 ligands *Cxcl9* and *Cxcl10* in B16-F10 tumors of HFD-fed mice (Fig. 2 F). IFN-γ promotes the infiltration of the tumor with leukocytes, including CD8 T cells, by driving the secretion of CXCL9 and CXCL10 (Groom and Luster, 2011). Interestingly, injection of recombinant IFN-γ beside the tumor was able to restore *Cxcl9* but not *Cxcl10* expression in B16-F10 tumors (Fig. S1 B). Together, these results suggest that obesity reduces the number of infiltrating immune cells, at least in part, by suppressing the expression of molecules involved in tumor trafficking and homing.

### Obesity impairs CD8 T cell effector function in tumors

**Figure S2.**
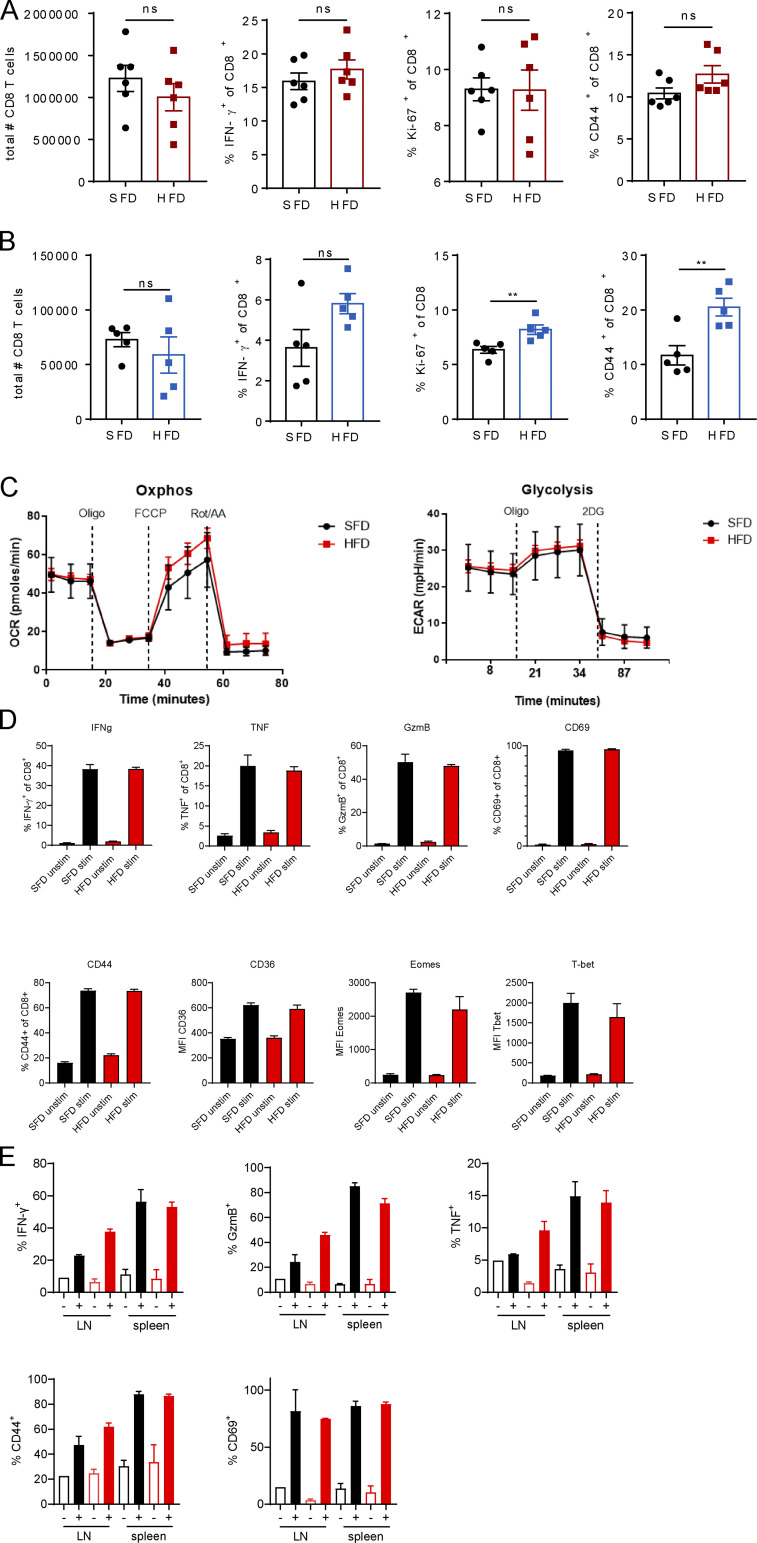
**CD8 T cell activation in dLNs is not impaired in obese mice. (A and B)** C57BL/6 mice were fed an SFD or HFD, and MC38 (A) or B16-F10 (B) tumor cells were injected s.c. Results depict flow cytometric analysis of CD8^+^ T cells in dLNs of tumor-bearing mice. Data are shown as individual mice and mean ± SEM; unpaired Student’s *t* test. **, P < 0.01 for three independent experiments for each tumor model. **(C and D)** CD8 T cells isolated from SFD-fed (*n* = 3; pooled) or HFD-fed (*n* = 3; pooled) mice were activated with anti-CD3/anti-CD28 and analyzed by Seahorse flux assay (C) or flow cytometry (D). Oxphos, oxidative phosphorylation. **(E)** CD8 T cells isolated from dLNs or spleens from SFD-fed (black; *n* = 3 technical replicates from 7 pooled mice) or HFD-fed (red; *n* = 3 technical replicates from 7 pooled mice) mice were activated with anti-CD3/anti-CD28 (+) or unstimulated (−) and analyzed by flow cytometry. Representative data from at least two independent experiments.

**Figure S3.**
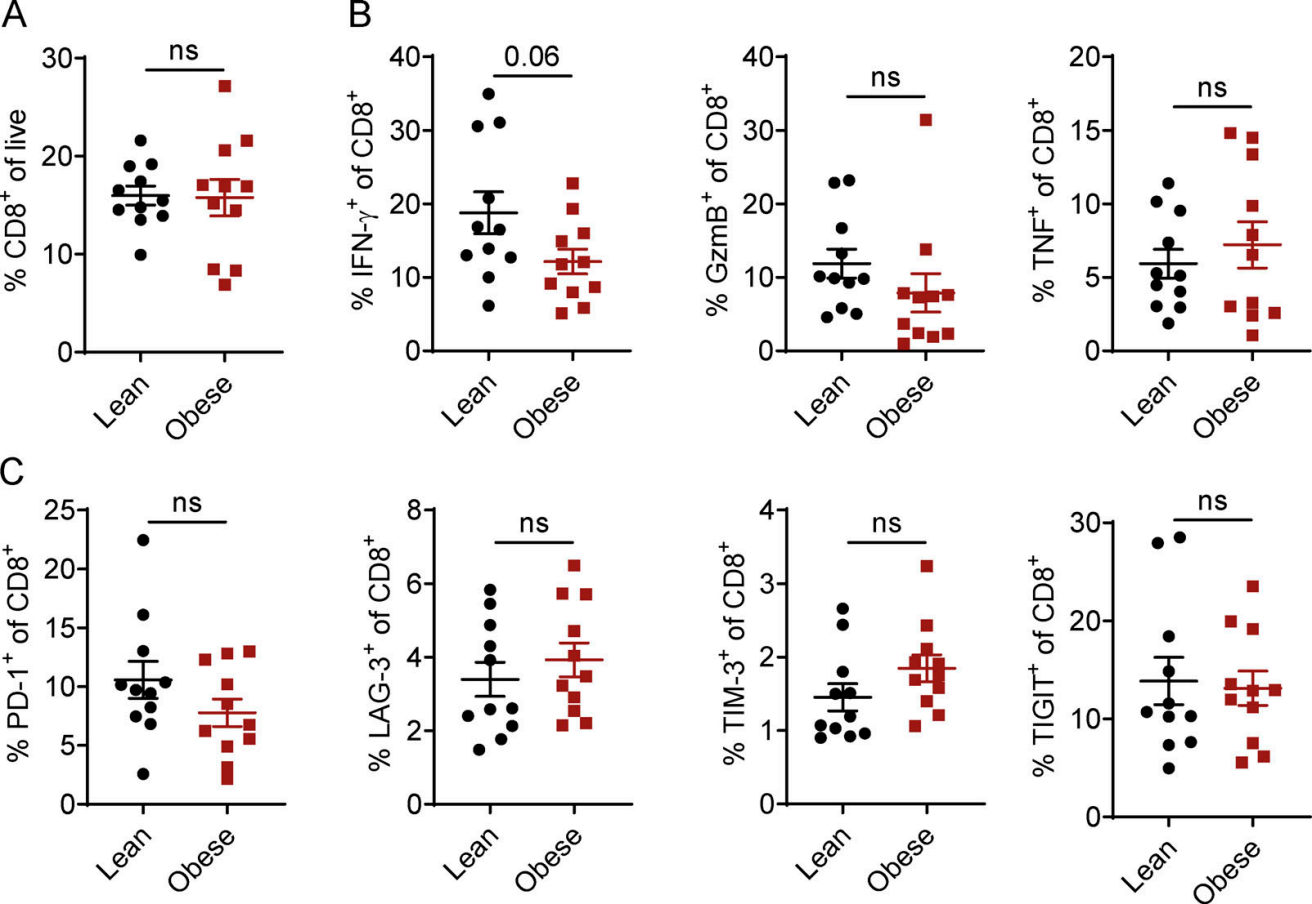
**Activation profile of human CD8 T cells from lean and obese donors. (A–C)** Flow cytometric analysis of CD8^+^ T cells in PBMCs of lean (*n* = 11) or obese (*n* = 11) donors. PBMCs were either stained directly (A and C) or stimulated with PMA, ionomycin, and BFA for 3.5 h before staining (B). Data are shown as individual donors and mean ± SEM; unpaired Student’s *t* test.

**Figure 3.**
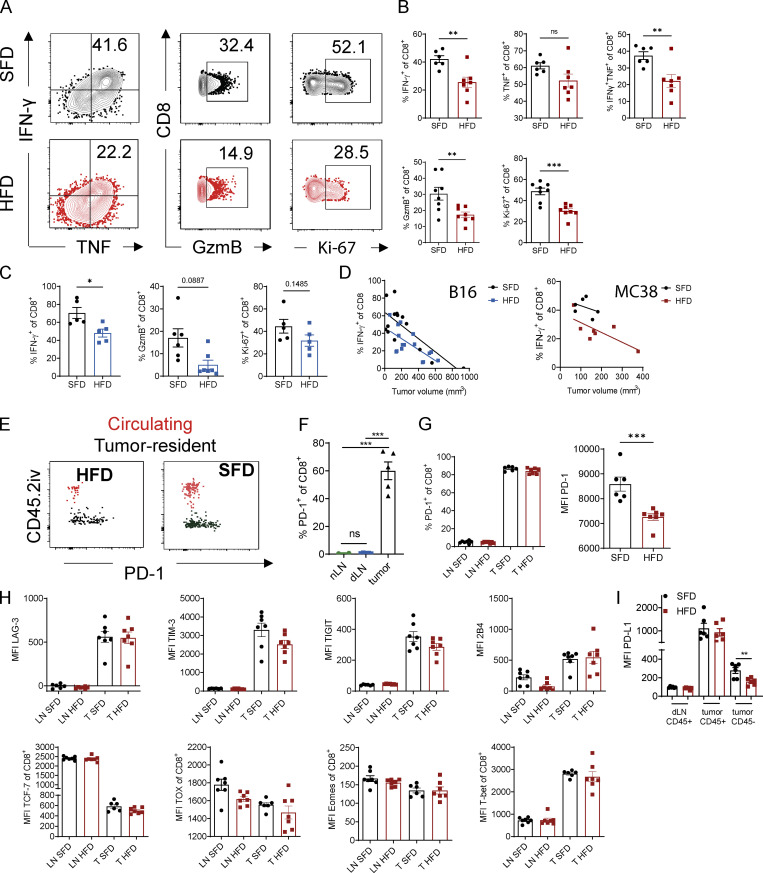
**HFD-induced obesity impairs CD8 T cell effector function in tumors independent of PD-1 expression. (A and B) **Flow cytometric analysis of intracellular IFN-γ, TNF, GzmB, and Ki-67 expression by CD8^+^ T cells in MC38 tumors of SFD-fed (*n* = 6) and HFD-fed (*n* = 7) mice. Results are shown as representative FACS plots (A) and percentage of cells (B). **(C)** Flow cytometric analysis of intracellular IFN-γ, GzmB, and Ki-67 expression by CD8^+^ T cells in subcutaneous B16-F10 tumors (*n* = 5 or 6 mice per group). Results are shown as percentage of cells. **(D)** Correlation between tumor volume and IFN-γ expression in CD8 T cells. Lines represent simple linear regression for each group. Pooled data from three experiments in B16 model (*n* = 14–16 mice per group) and one representative experiment for MC38 model (*n* = 6 mice per group). **(E)** Exemplary FACS plot of PD-1 expression on B16-F10 tumor-resident CD8^+^ T cells (CD45.2iv^−^; black) and tumor-circulating CD8 T cells (CD45.2iv^+^; red). **(F)** Flow cytometric analysis of PD-1 expression on CD8^+^ T cells from naive lymph nodes (nLNs), tumor dLNs, and MC38 tumors. **(G)** Flow cytometric analysis of PD-1 expression on CD8^+^ T cells from SFD- and HFD-fed mice in dLNs and MC38 tumors. **(H)** Flow cytometric analysis of surface markers and transcription factors on CD8 T cells from LNs and MC38 tumors. **(I)** Flow cytometric analysis of PD-L1 expression on CD45^+^ and CD45^−^ cells in dLNs and MC38 tumors from SFD- and HFD-fed mice. Data are shown as individual mice (dots) and mean ± SEM. **(B, C, F, and G)** Unpaired Student’s *t* test. **(E)** One-way ANOVA. *, P < 0.05; **, P < 0.01; ***, P < 0.001. This experiment was performed three times (B16 model) and at least twice for each graph (MC38 model).

**Figure S4.**
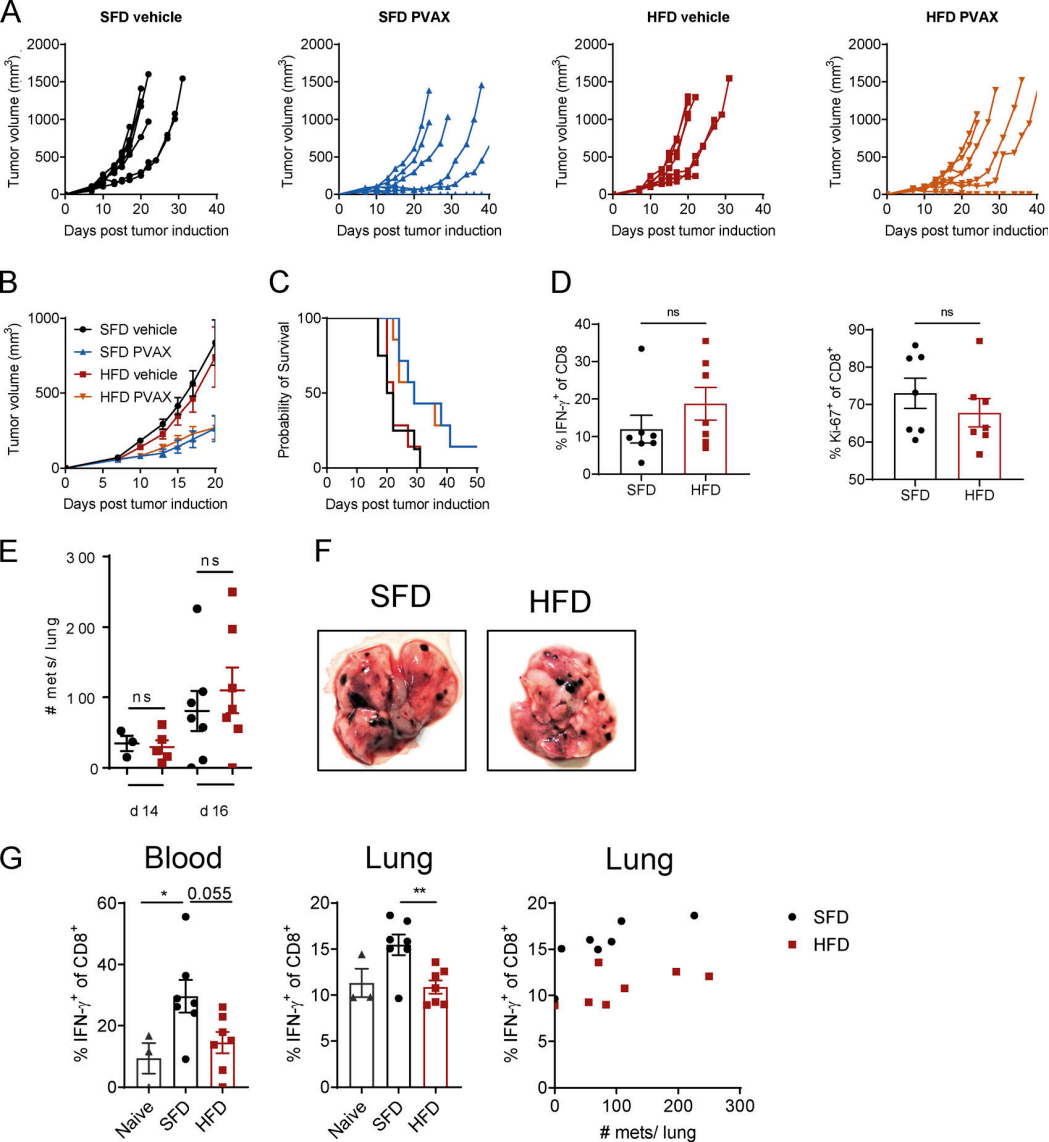
**Effect of HFD-induced obesity in the B16-F10 metastatic model. (A–C)** BALB/c mice were fed an SFD or HFD for 8 wk, and mice were injected s.c. with CT26 tumor cells (*n* = 7 or 8 mice per group; experiment was performed twice). On days 2 and 9, mice were injected i.p. with anti–PD-1 (200 µg/mouse), and, on days 3 and 10, mice were injected s.c. with a vaccine (hs/irr CT26 + R848) peritumorally. Figure depicts mean tumor growth ± SEM (A), percentage survival (i.e., mouse reached experimental endpoint; B), and individual tumor growth curves (C). **(D)** Tumor-infiltrating CD8 T cells were analyzed by flow cytometry. **(E–G)** C57BL/6 mice were fed an HFD or SFD for 8 wk, and mice were injected i.v. with B16-F10 tumor cells (combined data from two experiments; *n* = 3–7 per group). Graph shows number of lung metastases (mets) on days 14 and 16 after tumor injection (A) and exemplary images of lungs from SFD- and HFD-fed mice (B). **(C)** Flow cytometric analysis of IFN-γ expression by CD8^+^ T cells in the blood and lungs of non–tumor-bearing (naive) or SFD- or HFD-fed tumor-bearing mice. Data are shown as individual mice and mean ± SEM; unpaired Student’s *t* test (E and D) and one-way ANOVA (C). *, P < 0.05; **, P < 0.01.

**Figure 4.**
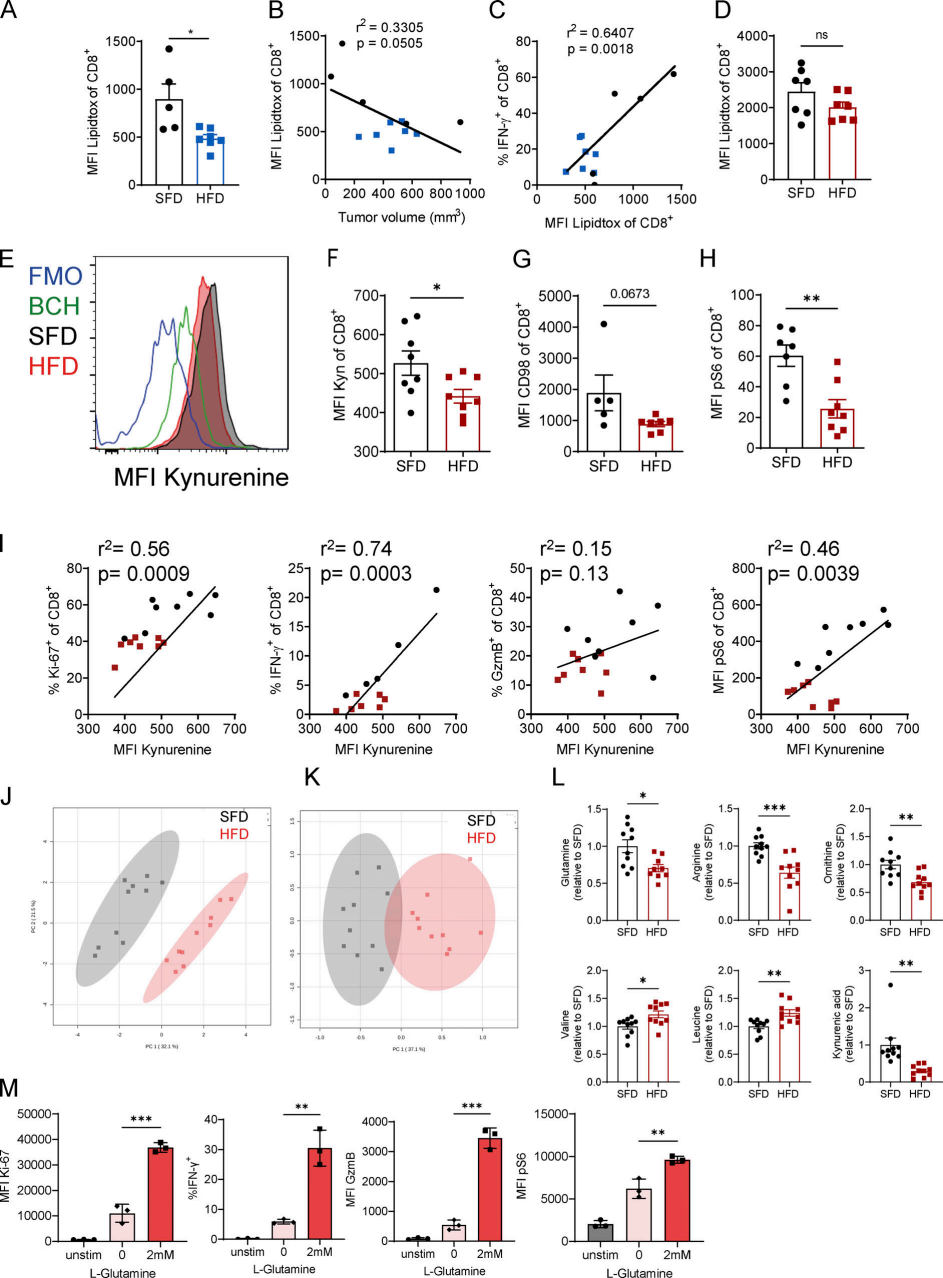
**Obesity-induced functional defects in CD8 T cells are associated with impaired amino acid metabolism. (A–D)** C57BL/6 mice were fed an HFD or SFD, and B16-F10 (A–C; *n* = 5 SFD, *n* = 7 HFD) and MC38 (D; *n* = 7 per group) tumors were injected. LipidTOX uptake by CD8^+^ T cells from tumors was analyzed by flow cytometry. Graphs depict MFI (A and D), correlation of LipidTOX versus tumor volume (B), and correlation of LipidTOX versus IFN-γ expression by CD8^+^ T cells (C). **(E–I)** C57BL/6 mice were fed an HFD (*n* = 8) or SFD (*n* = 8) for 6 wk, and MC38 tumors were injected s.c. On day 12 after tumor inoculation, tumors were dissected and stained for flow cytometry. **(E and F)** Representative graph, and quantification of kynurenine uptake by CD8^+^ T cells in tumors. Cells were treated with the system L blocker BCH or not treated with kynurenine (fluorescence minus one control [FMO]) as negative controls. **(G and H)** MFI or frequency of kynurenine, CD98, and pS6 in CD8^+^ T cells. **(I)** Correlation between kynurenine uptake and expression of intracellular molecules in CD8 T cells. Black dots indicate SFD-fed mice, and red dots indicate HFD-fed mice. **(J–L)** Serum taken from male SFD-fed (*n* = 10) or HFD-fed (*n* = 10) mice was analyzed by mass spectrometry for metabolite composition. Data are represented as fold change over SFD. Pooled data from two experiments. **(M)** CD8 T cells isolated from naive spleens were activated with anti-CD3/anti-CD28 in the presence or absence of L-glutamine, and expression of Ki-67, IFN-γ, GzmB, and pS6 was analyzed by flow cytometry. Experiment was performed twice (*n* = 3 technical replicates). **(A, D, F–H, and L) **Unpaired Student’s *t* test. **(B, C, and I) **Simple linear regression. **(M) **One-way ANOVA. *, P < 0.05; **, P < 0.01; ***, P < 0.001.

The control of tumor growth by T cells is largely dependent on their effector function and the secretion of effector molecules, including the cytokine IFN-γ and the cytolytic protease granzyme B (GzmB). Prior to tumor infiltration, however, T cells get primed in draining lymph nodes (dLNs), where they undergo clonal expansion in response to recognition of their cognate antigen. To investigate whether the reduction in tumor infiltration in obesity was associated with impaired T cell priming in the LNs, we analyzed T cell activation in dLNs of B16-F10 and MC38 tumor-bearing mice. In both models, we did not observe any differences in the total number of CD8 T cells or CD8 T cell IFN-γ production, proliferation, or expression of the activation marker CD44 in obese mice (Fig. S2, A and B). Interestingly, CD8 T cells expressed higher levels of Ki-67 and CD44 in obese than in lean B16-F10–bearing mice (Fig. S2 B). To further investigate whether feeding mice an HFD affects the ability of CD8 T cells to become activated, we isolated splenic CD8 T cells from lean and obese mice and activated them with anti-CD3/anti-CD28. By measuring the oxygen consumption rate (OCR) and extracellular acidification rate (ECAR), we found no differences in metabolic fitness between splenic CD8 T cells from lean or obese mice (Fig. S2 C). Moreover, outside of the tumor, splenic CD8 T cells from lean and obese mice secreted similar levels of IFN-γ, TNF, and GzmB and expressed similar levels of CD69, CD44, CD36, Eomes, and T-bet (Fig. S2 D). In addition, we isolated CD8 T cells from dLNs and spleens from MC38 tumor-bearing mice and activated them through their TCR. Again, expression levels of IFN-γ, TNF, GzmB, CD69, and CD44 were not significantly altered between lean and obese mice (Fig. S2 E). Thus, we did not observe a global defect in CD8 T cell function in obesity in mice. In human peripheral blood mononuclear cells (PBMCs), we did not detect significant differences in CD8 T cell frequency, GzmB, TNF, and IFN-γ expression between lean donors and donors with obesity, although there was a trend of reduced IFN-γ and GzmB expression in obesity (Fig. S3, A and B). We then assessed the effector function of CD8 T cells inside the tumors of lean and obese mice. In the MC38 model, obesity led to a reduction in IFN-γ^+^, double-expressing IFN-γ^+^TNF^+^, and GzmB^+^ CD8 T cells (Fig. 3, A and B). Moreover, CD8 T cells proliferated less in obese than in lean tumors as measured by Ki-67 expression (Fig. 3, A and B). Similar results were obtained for CD8 T cells from B16-F10 tumor-bearing mice (Fig. 3 C). To investigate whether these functional differences were simply due to differences in tumor size between lean and obese mice, we analyzed the expression of IFN-γ, as a functional marker, across the different tumor sizes. Interestingly, the expression of IFN-γ decreased with increasing tumor size in both lean and obese mice in the B16-F10 and MC38 models (Fig. 3 D). However, even at the same tumor size, expression of IFN-γ was lower in obese mice than in lean mice in both tumor models (Fig. 3 D). These findings suggest that obesity suppresses CD8 T cell function in the tumor not only due to the increased tumor size but also through additional mechanisms. We next assessed the effects of obesity on tumor growth in additional tumor models. In contrast to the B16-F10 or MC38 models, no differences in tumor growth or CD8 T cell IFN-γ or Ki-67 expression were observed in the CT26 tumor model, which is grown in BALB/c mice (Fig. S4, A–D), suggesting that the CT26 model is not susceptible to HFD-induced T cell dysfunction. In the metastatic B16-F10 tumor model, although HFD was not associated with increased metastatic foci in the lung (Fig. 4, E and F), there was a reduction in IFN-γ^+^ CD8 T cells in the blood and lungs compared with lean mice that was independent of the number of metastases in the lung (Fig. S4 G). Combined, these findings suggest that obesity does not impair baseline activation of CD8 T cells but suppresses T cell infiltration and, in addition, leads to functional defects in CD8 T cells in multiple tumor models, which may contribute to enhanced tumor growth.

Because CD8 T cells were functionally impaired in tumors of obese mice, we investigated the underlying mechanisms. PD-1 is an immune checkpoint that is frequently up-regulated on dysfunctional T cells in the tumor (Iwai et al., 2002; Pauken and Wherry, 2015). In line with previous reports, we found that PD-1 was significantly up-regulated when CD8 T cells entered the B16-F10 tumor tissue of both lean and obese mice compared with CD8 T cells in the tumor circulation (Fig. 3 E). Moreover, CD8 T cells expressed significantly higher levels of PD-1 in tumors than in naive LNs and dLNs (Fig. 3 F). While there were no significant differences in the frequency of PD-1^+^ CD8 T cells in dLNs or tumors between lean and obese mice, expression levels of PD-1 measured by mean fluorescence intensity (MFI) were significantly lower in CD8 T cells of obese mice (Fig. 3 G). To further investigate the exhaustion state of CD8 T cells in obese mice, we measured the expression of additional immune checkpoints and transcription factors associated with T cell exhaustion. Similar to PD-1, the immune checkpoints LAG-3, TIM-3, TIGIT, and 2B4 were up-regulated in CD8 T cells in tumors compared with dLNs but were not differentially expressed between lean and obese mice (Fig. 3 H). Moreover, we did not see any significant differences in the expression of the transcription factors TCF-7, TOX, Eomes, or T-bet in CD8 T cells of lean and obese mice (Fig. 3 H). We next measured the expression levels of immune checkpoints on CD8 T cells from PBMCs of lean human donors and individuals with obesity. Again, we found no significant differences in the expression of the immune checkpoints PD-1, LAG-3, TIM-3, and TIGIT between the two groups (Fig. S3 C). Taken together, although there was a trend of decreased function in intratumoral CD8 T cells in obesity, it does not appear to be due to checkpoint expression. Moreover, these findings suggest that obesity does not accelerate the classical T cell exhaustion program seen in CD8 T cells in tumors. Suppression of T cells through PD-1 is dependent on the expression of and interaction with its ligands, such as PD-L1; thus, we investigated whether PD-L1 expression was altered in obesity. In dLNs and tumors, CD45^+^ cells (leukocytes) in lean and obese mice expressed the same density of PD-L1 (Fig. 3 I). Interestingly, CD45^−^ cells (tumor cells/stroma) expressed lower levels of PD-L1 in obese mice than in lean mice (Fig. 3 I). Combined, these findings suggest that the functional defects in CD8 T cells in tumors of obese mice are not a consequence of increased immune checkpoint expression in the tumor microenvironment (TME); rather, they suggest that obesity promotes an “immune-cold” tumor phenotype through mechanisms other than accelerating immune checkpoint–driven T cell exhaustion (Bindea et al., 2014).

### Obesity-induced functional defects in CD8 T cells are associated with impaired amino acid metabolism

We have previously shown that NK cells become dysfunctional in obese mice and in people with obesity due to increased lipid uptake leading to metabolic paralysis (Michelet et al., 2018). Thus, we investigated whether a similar mechanism led to CD8 T cell dysfunction in obesity. We measured the lipid content of intratumoral CD8 T cells by LipidTOX staining and found that the LipidTOX MFI was significantly reduced in CD8 T cells in B16-F10 tumors from obese mice compared with lean mice (Fig. 4 A). The decrease in CD8^+^ LipidTOX was associated with an increase in tumor size, and LipidTOX staining positively correlated with IFN-γ production (Fig. 4, B and C). In the MC38 model, no differences in LipidTOX fluorescence intensity in CD8 T cells in tumors were observed between lean and obese mice (Fig. 4 D). These results suggest that CD8 T cell dysfunction in obesity is not due to lipid accumulation in the cells, as previously reported for NK cells (Michelet et al., 2018). Rather, lipid metabolism has been shown to be crucial for the metabolic programming and effector function of memory CD8 T cells (O’Sullivan et al., 2014), and thus a decrease in LipidTOX as seen in the B16-F10 model could be associated with suppressed CD8 T cell activation in obesity. We next investigated whether obesity altered cellular metabolism of CD8 T cells, which is intrinsically linked to their effector function. Activated CD8 T cells increase their metabolic rate, which is regulated by mammalian target of rapamycin (mTOR), HIF1α, and c-Myc and is dependent on amino acid uptake (Loftus and Finlay, 2016). CD8 T cell activation requires the activity of the amino acid transporter SLC7A5 which can be measured through the uptake of the fluorescent metabolite kynurenine (Sinclair et al., 2018; Sinclair et al., 2013). We found that CD8 T cells in MC38 tumors of obese mice had significantly reduced SLC7A5 activity compared with lean mice (Fig. 4, E and F). The SLC7A5 inhibitor 2-aminobicyclo-(2,2,1)-heptane-2-carboxylic acid (BCH) was used as a negative control for fluorescence in the absence of SLC7A5 activity (Fig. 4 E). Moreover, expression of CD98, which forms a complex with multiple amino acid transporters, including SLC7A5, was reduced on CD8 T cells from tumors of obese mice (Fig. 4 G). Phosphorylation of S6, which is downstream of mTOR signaling, was also significantly reduced in CD8 T cells of obese mice compared with lean mice (Fig. 4 H). Moreover, the expression of Ki-67, IFN-γ, and pS6 positively correlated with kynurenine uptake, confirming that SLC7A5 activity is associated with CD8 T cell effector function (Fig. 4 I). Having shown differences in amino acid uptake on a cellular level, we next investigated the link between obesity and metabolic remodeling at the systemic level. We analyzed the metabolome in the serum of lean and obese mice and found that SFD- and HFD-fed mice formed two very distinct clusters (Fig. 4 J). When only screening for amino acids, SFD- and HFD-fed mice again separated very strikingly (Fig. 4 K). We found that the abundance of multiple amino acids and their metabolic products was significantly altered in obesity, including glutamine, which was reduced in HFD-fed mice (Fig. 4 L). Glutamine is essential for CD8 T cell function and a substrate for SLC7A5 (Sinclair et al., 2018), suggesting that low levels of glutamine could impair CD8 T cell function in obesity. Indeed, when resubstituting glutamine to splenic CD8 T cells in vitro, we were able to restore CD8 T cell proliferation and expression of IFN-γ, GzmB, and pS6 (Fig. 4 M). These findings suggest that obesity leads to systemic metabolic remodeling, which potentially contributes to CD8 T cell functional defects.

### Immunotherapy protects from tumor growth in lean and obese mice

**Figure 5.**
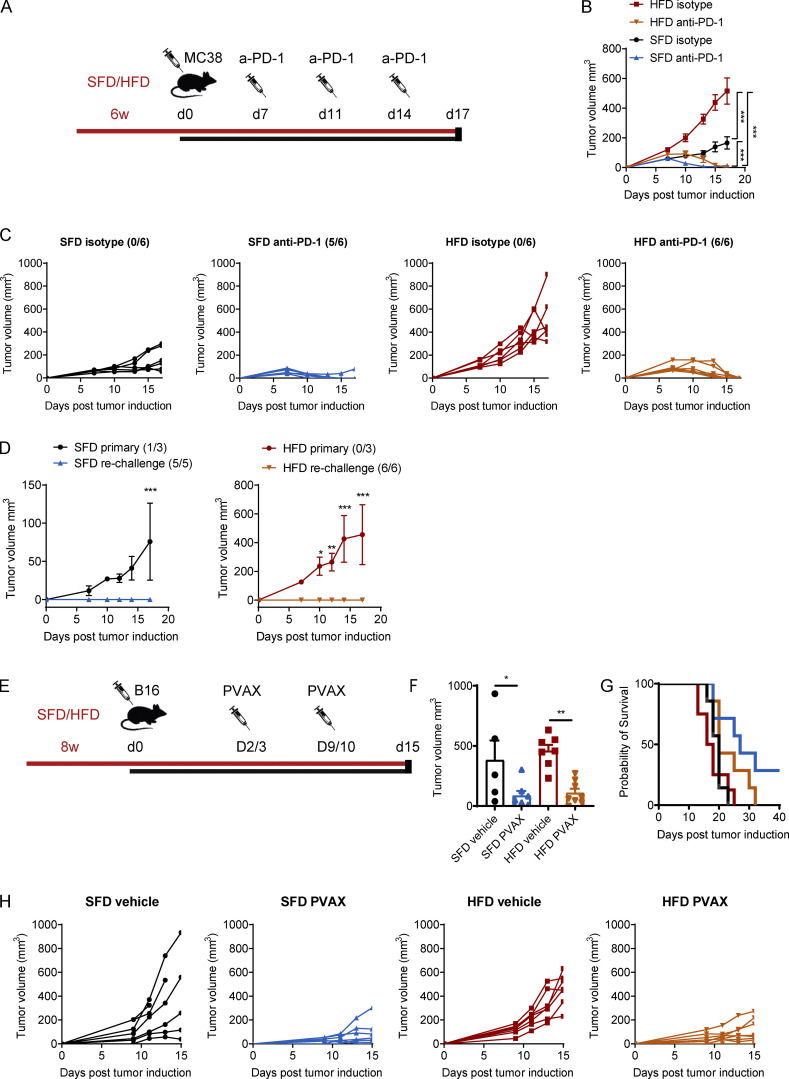
**Immunotherapy protects from tumor growth in lean and obese mice. (A–D)** C57BL/6 mice were fed an HFD or SFD for 6 wk, and MC38 tumors were injected. On days 7, 11, and 14, mice were injected i.p. with anti–PD-1 (200 µg/mouse). Figure depicts injection scheme (A), mean tumor growth ± SEM, two-way ANOVA (B), and individual tumor growth curves (C). **(D)** Mice that previously rejected tumors were rechallenged with MC38 tumor cells and compared with SFD- or HFD-fed mice that had not been injected with tumor cells previously (primary). Graphs depict mean tumor growth ± SEM (two-way ANOVA). Numbers in brackets indicate the number of mice that rejected the tumor out of the total mice in the group (*n* = 6 per group; experiment was performed four times). **(E–H)** C57BL/6 mice were fed an HFD or SFD for 8 wk, and B16-F10 tumors were injected. On days 2 and 9, mice were injected i.p. with anti–PD-1 (200 µg/mouse), and, on days 3 and 10, mice were injected s.c. with a vaccine (hs/irr B16-F10 + R848) peritumorally. Experiment was performed twice (*n* = 7 or 8 per group). Figure depicts injection scheme (E); mean tumor volume ± SEM on day 15; dots represent individual mice; one-way ANOVA (F); percentage survival (i.e., mouse reached experimental endpoint; G); and individual tumor growth curves (H). *, P < 0.05; **, P < 0.01; ***, P < 0.001.

We have demonstrated that obesity leads to more rapid tumor progression in multiple tumor models, which is associated with reduced TIL infiltration and suppressed CD8 T cell metabolism and effector function. Immunotherapy has been shown to increase the pool of activated CD8 T cells and reverse metabolic dysfunction (Barber et al., 2006; Bengsch et al., 2016; Chang et al., 2015). Thus, we next sought to determine whether obesity-induced immunosuppression affects the efficacy of cancer immunotherapy or whether ICB therapy could potentially restore CD8 T cell function in obesity. Mice were fed an SFD or HFD, and MC38 tumors were injected, followed by anti–PD-1 injection, on days 7, 11, and 14 after tumor inoculation (Fig. 5 A). Once again, tumors in HFD-fed mice grew significantly quicker than in SFD-fed mice (Fig. 5 B). Injection with anti–PD-1 led to complete tumor regression in 80% of SFD-fed mice and 100% of HFD-fed mice (Fig. 5 C). Thus, obesity did not impair the efficacy of anti–PD-1 therapy in the MC38 model. Mice that rejected the tumors were rechallenged with MC38 tumor cells 2 mo after tumor clearance. Upon rechallenge, both lean and obese mice that had previously cleared the tumor were completely protected from tumor growth (Fig. 5 D), suggesting the presence of immune memory, which was not affected by obesity. We next investigated the effects of obesity on immunotherapy in additional tumor models, using mice transplanted with B16-F10 or CT26 tumors. B16-F10 and CT26 tumors do not respond to anti–PD-1 alone; thus, these mice received a combination therapy with anti–PD-1 and an experimental vaccine (Dyck et al., 2016). Mice were treated with anti–PD-1 on days 2 and 9 after tumor induction and vaccinated with heat-shocked/γ-irradiated (hs/irr) tumor cells and the TLR7 agonist R848 on days 3 and 10 (Fig. 5 E). Similar to the MC38 model, the anti-PD-1 vaccine combination (PVAX) significantly reduced B16-F10 as well as CT26 tumor growth and prolonged survival in SFD- and HFD-fed mice (Fig. 5, F–H; and Fig. S4, A–C). These findings suggest that although HFD-induced obesity enhances tumor growth, it does not impair the efficacy of immunotherapy using different immunotherapy regimens including anti–PD-1 and cancer vaccines.

### Obesity-associated defects in CD8 T cell function can partially be restored with immunotherapy

**Figure 6.**
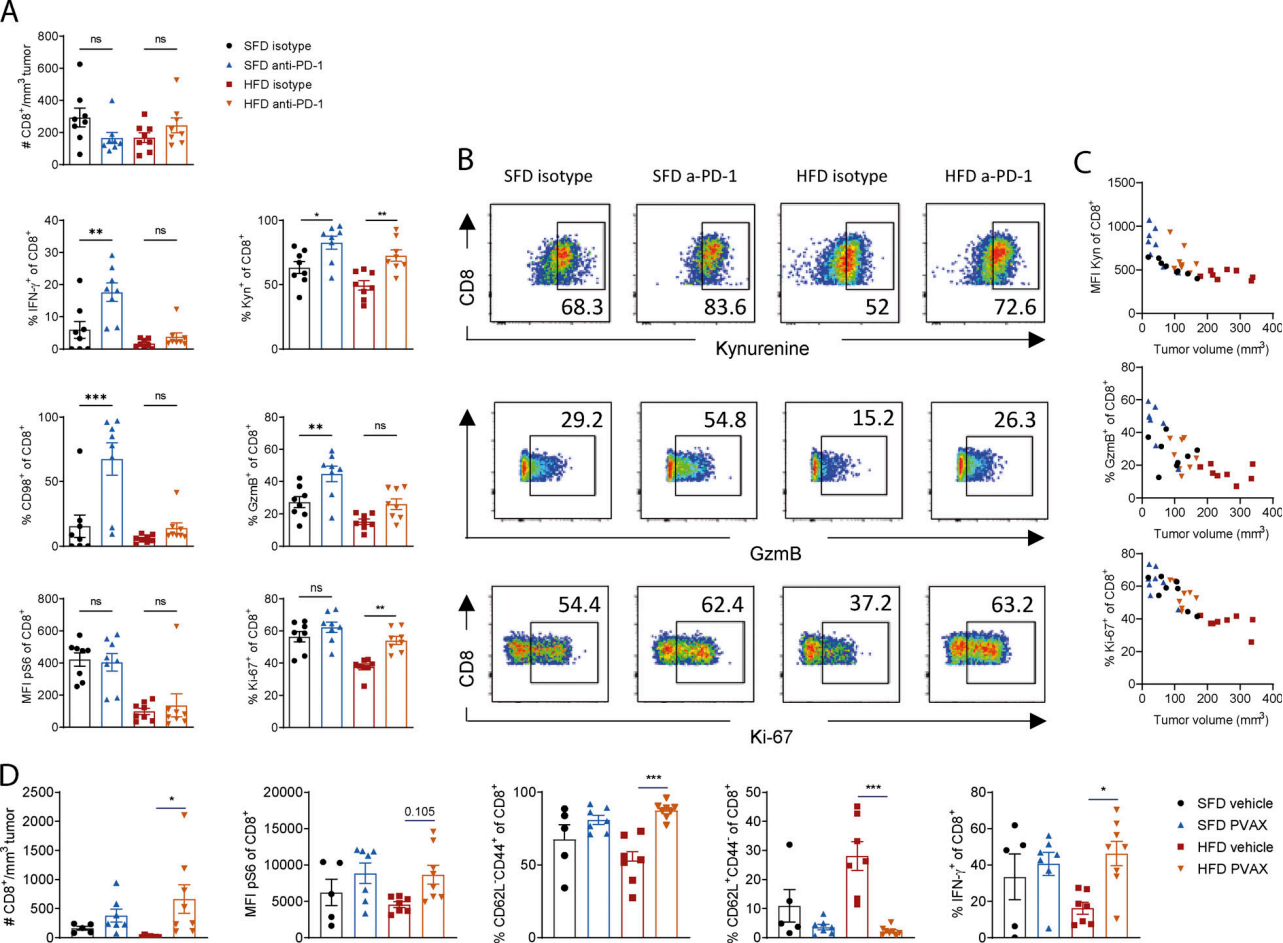
**Immunotherapy partially restores CD8 T cell function in lean and obese mice. (A–C) **C57BL/6 mice were fed an HFD or SFD for 6 wk, and MC38 tumors were injected as in Fig. 5 A. On day 10, mice were injected i.p. with anti–PD-1 (200 µg/mouse), and tumors were analyzed on day 12. Data are shown as individual mice (dots; *n* = 7 or 8 per group) and mean ± SEM (A), representative FACS plots (B), and correlation between tumor size and kynurenine, Ki-67, or GzmB expression (C). Experiment was performed three times. **(D)** C57BL/6 mice were fed an HFD or SFD for 8 wk, and B16-F10 tumors were injected as in Fig. 5 E. On days 2 and 9, mice were injected i.p. with anti–PD-1 (200 µg/mouse), and, on days 3 and 10, mice were injected s.c. with a vaccine (hs/irr B16-F10 + R848) peritumorally. On day 15, tumors were analyzed by flow cytometry. Figure depicts flow cytometric analysis of CD8^+^ T cells in the tumors. Data are shown as individual mice (dots; *n* = 5–7 per group) and mean ± SEM. Experiment was performed twice. Significance was calculated using one-way ANOVA. *, P < 0.05; **, P < 0.01; ***, P < 0.001.

We next analyzed the infiltration and function of CD8 T cells in the tumors after ICB. To get enough MC38 tumor mass to be able to analyze infiltrating immune cells, we delayed the therapy until tumors were fully established, injected one dose of anti–PD-1 on day 10 after tumor induction, and analyzed TILs 2 d after therapy. At this early time point, anti–PD-1 treatment restored several, but not all, defects in CD8 T cells. In both SFD- and HFD-fed mice, there was no significant difference in the number of infiltrating CD8 T cells (Fig. 6 A). In lean mice, anti–PD-1 treatment significantly increased IFN-γ, kynurenine uptake, and CD98 and GzmB expression but not pS6 or Ki-67 expression (Fig. 6, A and B). In obese mice, CD8 T cells significantly increased kynurenine uptake, proliferated more, and showed a trend toward increased GzmB expression, but no significant differences in IFN-γ, CD98, or pS6 were detected at this time point (Fig. 6, A and B). Interestingly, the increase in kynurenine uptake, proliferation, and GzmB expression after PD-1 blockade was associated with a reduction in tumor size in lean and obese mice, but levels of kynurenine uptake and GzmB exceeded those of control mice at the same tumor size (Fig. 6 C). This suggests that the increase in some effector molecules in CD8 T cells after anti–PD-1 is not due solely to a reduction in tumor size. In B16-F10 tumor-bearing mice, treatment with PVAX led to potent restoration of a proinflammatory CD8 phenotype in lean and obese mice on day 15 after two doses of therapy, including increased CD8 T cell infiltration, IFN-γ production, and pS6 expression in CD8 T cells from obese mice compared with obese mice treated with a vehicle (Fig. 6 D). Moreover, there was a significant decrease in the proportion of CD8 T cells with a naive phenotype (CD62L^+^CD44^−^) and an increase in the effector memory–like phenotype (CD62L^−^CD44^+^) in tumors of obese mice after immunotherapy (Fig. 6 D). These findings demonstrate that the reduction in tumor growth after immunotherapy is associated with a partial restoration of CD8 T cell function in lean and obese mice. Importantly, even though only some of the defects in CD8 T cells were restored at the time points shown here, the overall effect of immunotherapy was sufficient to induce tumor regression in the MC38 model and slow down tumor growth in the B16 model.

### Effects of obesity on immune cell infiltration in human cancers

Obesity is a risk factor for several types of cancer, including endometrial cancer, colorectal cancer, and esophageal adenocarcinoma (De Pergola and Silvestris, 2013; Renehan et al., 2008). While altered hormone levels are implicated in driving obesity-associated tumorigenesis, little is known regarding whether defects in anti-tumor immune responses also contribute to increased cancer risk in obesity. To investigate the link between anti-tumor immunity and obesity, we investigated the immune profile of human endometrial cancers, which is the cancer type with the highest association with obesity (De Pergola and Silvestris, 2013).

**Figure 7.**
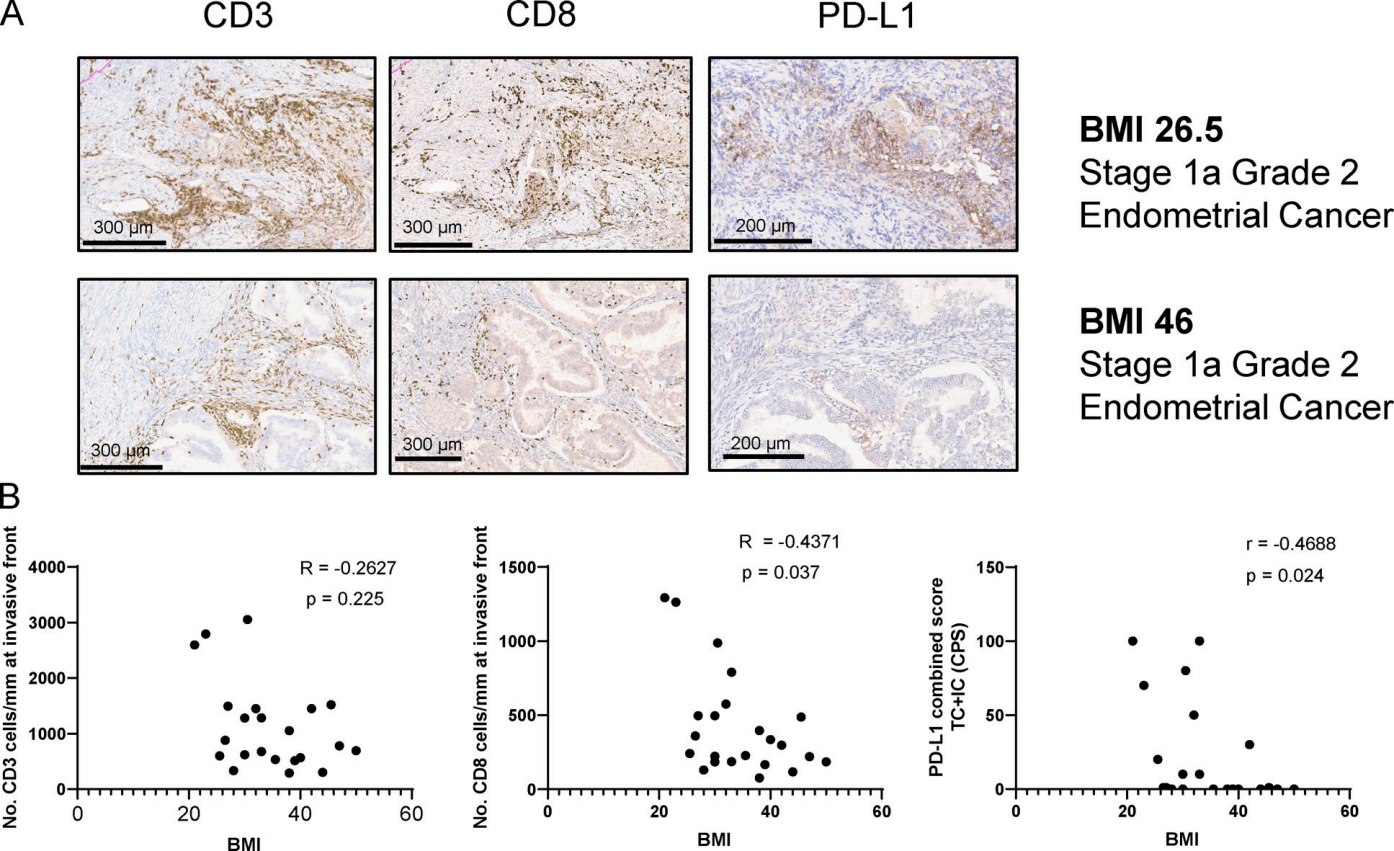
**Obesity is associated with impaired immune infiltration in humans with endometrial cancer.** Immune profiles of endometrial cancer tumors and IF in a blinded normal and obese patient study (*n* = 24). **(A)** Exemplary images of CD3, CD8, and PD-L1 immunohistochemical staining in IF of two stage 1a, grade 2 endometrial cancer patients (BMI 26.5 kg/m^2^ and BMI 46 kg/m^2^, respectively). **(B)** Correlation of CD3, CD8, and PD-L1 scores with patient BMI. Correlation was computed using nonparametric Spearman correlation.

To investigate the effects of obesity on immune infiltration in endometrioid endometrial cancer, we measured the expression of CD3, CD8, and PD-L1 in the tumor and invasive front (IF) in a blinded patient study involving a total of 24 patients with or without obesity. Tumor biopsies were immunostained for CD3, CD8, and PD-L1, and positive cell staining was calculated using algorithms for markup images. We found that expression of CD8 T cells was significantly reduced with increasing BMI within the IF of endometrial tumors (Fig. 7, A and B). We also observed a nonsignificant reduction of total CD3 expression with increasing BMI (Fig. 7, A and B). In addition, the PD-L1 combined score also significantly decreased with increasing BMI (Fig. 7 B).

**Figure S5.**
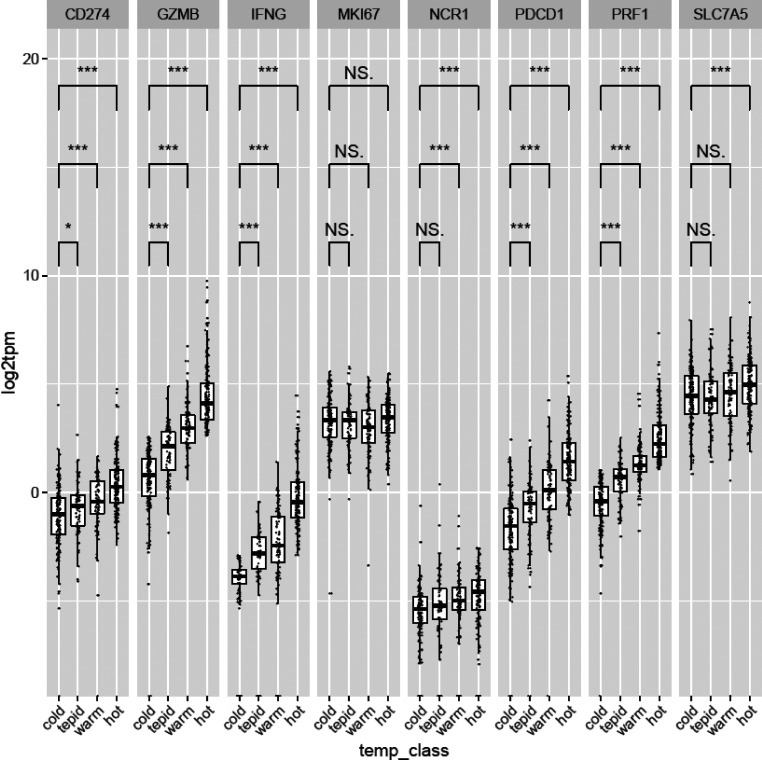
**Genes associated with tumor temperature classes.** Correlation of genes with tumor temperature classes. Patient samples were scored by their median expression value for IFN-γ, GzmB, and PRF1 (+0 for below median, +1 for above median). Scores were summed to give four temperature classes (0–3), named as “cold,” “tepid,” “warm,” and “hot,” respectively. Wilcoxon rank-sum tests were used to determine if the distribution of genes of interest was significantly different between each temperature class. *, P < 0.05; ***, P < 0.001.

Endometrial tumors from The Cancer Genome Atlas (TCGA) were next classified by temperature, in terms of immunoscore, ranging from cold to tepid, warm, and hot. The classification was based on their median GzmB, IFN-γ, and perforin expression, cold being associated with low and hot with high expression of GzmB, IFN-γ, and perforin. Interestingly, the expression of PD-1 and PD-L1 was significantly increased as tumors got hotter, in line with the findings that PD-1 and PD-L1 are up-regulated during T cell activation and infiltration (Fig. S5). Moreover, the expression of the amino acid transporter SLC7A5 was significantly higher in hot tumors than in cold tumors, supporting a role for SLC7A5 in anti-tumor immunity in human cancers (Fig. S5). Overall, these findings suggest that an increased BMI in endometrial cancer is associated with immune cell exclusion and a colder immune phenotype characterized by reduced T cell infiltration and PD-L1 expression.

**Figure 8.**
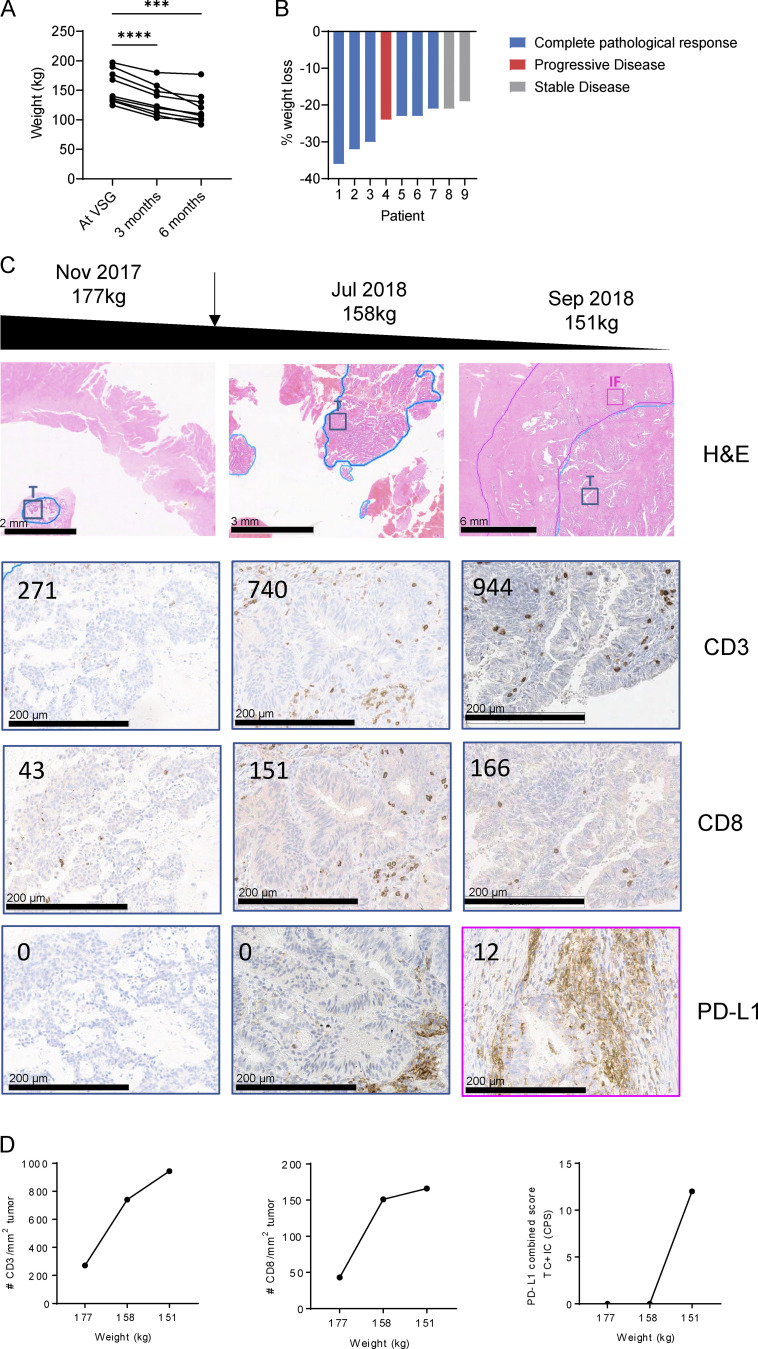
**Weight loss restores T cell infiltration in endometrial cancer. (A and B)** Patients with endometrial cancer (*n* = 9) were analyzed for weight loss (A) and cancer disease progression (B) after VSG. **(C and D)** Immune profile of tissue samples taken from a patient with endometrial cancer over the course of their treatment. The patient received VSG in April 2018, and samples were taken before and after VSG. **(C)** H&E (top panel) and IHC images of tumor tissue (T; blue) and IF (pink) stained for CD3, CD8, and PD-L1. Numbers in images represent the number of CD3^+^ or CD8^+^ T cells per mm^2^ tumor or PD-L1 combined score. **(D)** CD3 and CD8 infiltration and PD-L1 scores over the course of weight loss. Significance was calculated using one-way ANOVA (A). ***, P < 0.001; ****, P < 0.0001.

To further investigate a potential link between obesity and tumor growth in endometrial cancer, we assessed a consecutive cohort of patients who underwent metabolic surgery for weight loss. Metabolic surgery was performed after endometrial cancer diagnosis but before the surgery to remove the endometrial tumor. In all nine patients, vertical sleeve gastrectomy (VSG) led to significant weight loss over 6 mo (Fig. 8 A). Strikingly, this weight loss was associated with tumor regression in six patients and stable disease in two patients, while only one patient progressed (Fig. 8 B).

In one patient, we were able to obtain longitudinal tumor biopsies during the course of weight loss and characterize the immune infiltrate by histology. This patient with grade 1 endometrioid adenocarcinoma had a levonorgestrel intrauterine device (LNG-IUD) in situ but received no other systemic therapy. Endometrial biopsies were collected before and at 3 and 6 mo after VSG, before the tumor was then surgically removed 6 mo after bariatric surgery. Tumor biopsies were stained by immunohistochemistry for CD3, CD8, and PD-L1 at the IF and tumor. Before the surgery, the patient weighed 177 kg, and the tumor stained negative for PD-L1 and had low CD8 (43 CD8/mm^2^ tumor) and CD3 (271 CD3/mm^2^ tumor) counts (Fig. 8 C). As the patient lost weight over 6 mo, as a result of metabolic surgery, from 176 kg at the time of the surgery to 158 kg after 3 mo and 151 kg after 6 mo, the number of infiltrating CD3^+^ and CD8^+^ cells increased dramatically within the tumor (Fig. 8, C and D). In addition, PD-L1^+^ immune cells were detected in the tumor and IF at the time the patient had lost 15% of body weight (Fig. 8, C and D). These findings highlight that weight loss in endometrial cancer can lead to tumor regression, potentially by restoring immune cell infiltration.

## Discussion

Obesity is a major risk factor for 11 types of cancer (Kyrgiou et al., 2017), but the effect of obesity on the immune response within the tumor is largely unknown. Here, we report that obesity enhances tumor growth and reduces CD8 T cell infiltration, proliferation, and function in the tumor. The defects in CD8 T cell infiltration and effector function were associated with impaired chemokine expression and amino acid metabolism in obesity. Despite the suppressive effects of obesity, immunotherapy regimens using anti–PD-1 alone or in combination with an experimental cancer vaccine led to tumor rejection in lean and obese mice and partially restored a proinflammatory CD8 T cell phenotype. Moreover, in humans, obesity was associated with reduced CD8 T cell infiltration in endometrial cancers. Weight loss after metabolic surgery led to tumor regression in the majority of patients, and, in a case study, this cold phenotype was restored after weight loss, highlighting the suppressive yet reversible effects of obesity on anti-tumor CD8 T cell responses in mice and humans.

Obesity strongly enhanced tumor growth in the MC38 tumor model but only slightly in the B16-F10 model and not at all in the CT26 model. These differences are likely due to different immunogenicities in these models. The B16-F10 tumor cell line is highly aggressive and lowly immunogenic. It is not surprising that further suppression of the already suppressed immune system does not increase tumor growth, which is already at its maximum growth rate. On the other hand, MC38 is highly immunogenic, and its growth rate is limited by CD8 T cells. Ringel et al. (2020) recently showed that blocking CD8 T cells hugely accelerated MC38 tumor growth in vivo. Thus, MC38 cells are more affected by diet-induced systemic changes and suppression of the immune system. Lastly, no effect of an HFD on tumor growth or CD8 T cell function was observed in the CT26 model, suggesting that BALB/c mice are less susceptible to diet-induced changes of the immune system, and BALB/c mice have been reported to be protected from some aspects of obesity (Montgomery et al., 2013).

Despite the different susceptibilities to obesity-driven tumor progression, functional defects in CD8 T cells were found both in MC38 and B16-F10 tumors. The underlying causes of CD8 dysfunction are most likely a combination of several effects of the HFD on the tumor directly and on the affected cells of the immune system. First, we found that the number of immune cells in the tumor was reduced in obesity. At the same time, the expression of chemokines (*Cxcl9*,* Cxcl10*) and the chemokine receptor CXCR3, which are key for traffic to the tumor and extravasation, were highly down-regulated in obesity, suggesting that lower chemokine levels prevented effective recruitment of immune cells to the tumor site. Previous studies have reported aberrant chemokine expression in adipose tissue in obesity, with some chemokines increasing and others decreasing, demonstrating a chemokine imbalance in obesity (Huber et al., 2008; Wu et al., 2007). Our findings demonstrate that the obese environment also leads to altered chemokine secretion in the tumor leading to a reduction of CXCL9 and CXCL10 in obese tumors. Since CXCL9 and CXCL10 are IFN-γ–inducible chemokines (Van Raemdonck et al., 2015), lack of IFN-γ secretion by immune cells in the tumor could lead to a negative feedback loop, preventing further CD8 T cell recruitment. This is supported by our findings that injecting recombinant IFN-γ into the vicinity of the tumor restored Cxcl9 expression in the tumor, supporting the above hypothesis that a lack of IFN-γ contributes to impaired immune infiltration.

In addition to a reduction in immune cell infiltration, we found that CD8 T cells were functionally impaired within the tumors but not in the dLNs. Moreover, T cells isolated from the LNs and spleens of HFD-fed mice were still responsive to activation through their TCR. In contrast, the functional defects in the tumor were associated with metabolic dysfunction. Interestingly, we did not observe an increase in lipid uptake by CD8 T cells in obesity, unlike in NK cells (Michelet et al., 2018), suggesting that lipid uptake is not the cause of metabolic dysfunction. However, we found reduced activity of the amino acid transporter SLC7A5 in tumor-infiltrating CD8 T cells of obese mice. SLC7A5 has been shown to be essential for CD8 T cell activity and cytokine production (Sinclair et al., 2018, 2013). Previous studies have suggested that the TME can become deficient in levels of key metabolites, such as glucose, which is required for T cell function, although a recent paper shows that glucose is abundant and available to immune cells (Chang et al., 2015; Ho et al., 2015; Reinfeld et al., 2021). In addition, the TME can directly suppress T cell metabolism by creating areas of hypoxia as a result of uncontrolled angiogenesis and improper vascularization (Brown and Wilson, 2004). However, amino acid availability is arguably the most crucial metabolic gatekeeper that governs T cell metabolism and function by regulating both signaling transduction and protein synthesis (Sinclair et al., 2018, 2013). We found that feeding an HFD led to systemic remodeling of the metabolome, including major changes in amino acid composition and reduction in glutamine availability. The importance of amino acid availability for T cell function is highlighted by the fact that tumor cells can restrict T cell amino acid availability in multiple ways. Some tumors are dependent on glutamine, a key regulator of SLC7A5 activity (Sinclair et al., 2013), and directly compete with TILs for glutamine availability in the TME. In addition, tumor cells secrete amino acid–depleting substances such as the tryptophan-depleting enzyme indoleamine-pyrrole 2,3-dioxygenase (Munn and Mellor, 2016; Uyttenhove et al., 2003). Our findings suggest that a reduction in the uptake of amino acids contributes to the functional defects of CD8 T cells in tumors in obesity. Since an increasing tumor volume also leads to reduced nutrient and oxygen levels, direct effects of obesity on tumor growth, such as through hormones, could further contribute to the lack of nutrients available for immune cells in obesity. Overall, our findings suggest that ICB also works through metabolic reinvigoration of T cells, because blockade of PD-1 restored SLC7A5 activity in CD8 T cells in the tumors.

Recent reports suggested a role for immune checkpoints in obesity-driven tumor growth. Wang et al. (2019) reported an increase of leptin-induced PD-1 expression in obesity on CD8 T cells in humans, primates, and mice. Using the B16-F0 model, which is more immunogenic than B10-F10, they also found increased PD-1 expression on CD8 T cells in the tumor (Wang et al., 2019). In contrast, we did not observe increased expression of PD-1 on CD8 T cells in mice or humans. This is most likely because PD-1 expression in the tumor in SFD-fed mice already reached 90% in our experiments, which is in line with previous reports (Dyck et al., 2016; Juneja et al., 2017). Thus, there is a saturated expression with little room for increase, and, indeed, we even saw lower expression levels of PD-1 in CD8 T cells in obesity when looking at the MFI. Wang et al. found that, in the tumor, the level of PD-1 expression on CD8 T cells was considerably lower than in our findings (40% versus 80%). The rest of the study by Wang et al. focused on PD-1 expression in the livers of lean and obese mice without tumors and showed more PD-1 in the livers of obese mice. We did not investigate the PD-1 expression in the liver, but we found no differences in tumors or LNs of obese mice. Moreover, additional immune checkpoints, such as TIM-3, LAG-3, TIGIT, and EB4, and exhaustion markers, such as TOX, TCF-7, and Eomes, were not increased on CD8 T cells in obesity, suggesting that obesity does not accelerate classical T cell exhaustion. Furthermore, we have seen no increase in immune checkpoints on peripheral CD8 T cells in humans with obesity. We also found that reduced PD-L1 was associated with obesity in mice and humans. Our findings highlight that mechanisms independent of immune checkpoint expression led to a significant increase in tumor burden and contributed to CD8 T cell dysfunction in tumors of obese mice.

Our study is in line with a recent publication that showed HFD-induced metabolic remodeling and dysfunction of CD8 T cells in the tumor. Ringel et al. (2020) found that obesity accelerated MC38 tumor growth, which was associated with a reduction in CD8 T cell function. Interestingly, they showed that tumor cells and CD8 T cells differentially adapted to the HFD with only the tumor cells increasing their lipid uptake, while CD8 T cells became lipid starved. In contrast, Manzo et al. (2020) reported that CD8 T cells accumulated lipids in a pancreatic cancer model in a nonobese setting, leading to T cell exhaustion. Similarly to Ringel et al., we did not see an accumulation of lipids in CD8 T cells, highlighting that other mechanisms are at play in CD8 T cell dysfunction in obese tumors, and our data support impaired amino acid metabolism as a key factor, both systemically and cellularly, in CD8 T cells.

Despite the suppressive effects of obesity on anti-tumor immunity, we found that immunotherapy using anti–PD-1 alone or in combination with a cancer vaccine was effective in lean and obese mice and led to tumor rejection. Immunotherapy restored CD8 T cell infiltration and function and amino acid uptake. Although PD-1 expression was not altered in obesity, blockade of PD-1 led to complete tumor rejection of MC38 tumors in lean and obese mice. This observation suggests that separate mechanisms promote tumor growth versus those that are activated upon checkpoint inhibition. While tumor growth is, at least in part, enhanced through impaired T cell infiltration and metabolic dysfunction, blockade of PD-1 was able to override the tumor-promoting effects of obesity. This is an important observation, as it suggests that obesity-associated defects in CD8 T cells are reversible and that obesity-independent suppressive mechanisms, such as PD-1 engagement, remain the main drivers of T cell exhaustion in some tumor models. We did not observe a complete restoration of CD8 T cell responses with anti–PD-1, which could be due to the timing of the experiment, because we analyzed CD8 T cells shortly after one dose of anti–PD-1 therapy. Our results display early effects of checkpoint blockade, but it cannot be ruled out that additional functional markers get restored at a later time point, especially because anti–PD-1 therapy led to tumor rejection in the MC38 model. Moreover, other mechanisms, such as direct effects of anti–PD-1 on tumor cells (Kleffel et al., 2015) or myeloid cells (Strauss et al., 2020), could have played a role as well, suggesting that anti–PD-1 exerts its effect through multiple mechanisms. Our findings, however, are in line with several recent publications reporting that patients with obesity respond better to PD-1–based ICB than lean or underweight patients (Cortellini et al., 2019; McQuade et al., 2018; Naik et al., 2019; Wang et al., 2019). Because a significant proportion of patients does not respond to ICB (Park et al., 2018; Robert et al., 2015b), it is important to identify predictors of response. While obesity is a risk factor for many types of cancer and suppresses anti-tumor immunity, our study supports the emerging view that obesity is not a negative predictor of ICB success. This finding is consistent with the “obesity paradox” in cancer immunotherapy. However, we suggest that obesity and ICB have distinct mechanisms by which they affect tumor growth.

Studies correlating the efficacy of checkpoint blockade with obesity have been conducted in melanoma patients (Cortellini et al., 2019; McQuade et al., 2018; Naik et al., 2019). Melanoma was the first cancer type in which ICB using anti–PD-1 was demonstrated to induce a clinical response (Topalian et al., 2012) and was consequently approved for treatment (Iwai et al., 2017). However, whether it is linked to obesity is still under debate (Kyrgiou et al., 2017). Thus, it is largely unknown how obesity affects immune infiltration and immunotherapy in obesity-associated cancers such as endometrial cancer, which has a strong obesity link. Previous work identified increased circulating estrogen, insulin resistance, chronic inflammation, and adipokines as mechanisms by which obesity can promote endometrial cancer (Morice et al., 2016). In this study, we showed for the first time that impaired anti-tumor immunity is a novel link to be explored between obesity and increased endometrial cancer risk. We found that an increased BMI in endometrial cancer was associated with lower CD8, CD3, and PD-L1 expression. In part, this might be due to lower tumor mutational burden, because patients with high BMI are more likely to be in the microsatellite stable category, which leads to lesser recognition by immune cells because lesser amounts of neoantigens are expressed (Cohn et al., 2008). However, we found that in the same patient, weight loss after bariatric surgery increased immune cell infiltration and PD-L1 expression, suggesting that obesity also has a direct effect on anti-tumor immunity in endometrial cancer. These findings highlight a novel mechanism by which obesity might promote tumor development, and they have wider implications for therapy. For example, metabolic surgery and weight loss could potentially make patients more responsive to immunotherapy or other therapies. However, it is still unknown how weight or adiposity affects the efficacy of immunotherapy in obesity-associated cancers such as endometrial cancer, and larger studies are needed to study this link.

In conclusion, our findings demonstrate that the suppression of CD8 T cell anti-tumor immunity is a novel link between obesity and tumor progression. By amplifying CD8 T cell responses, such as through ICB or weight loss, a significant reduction in tumor growth was achieved in mice and humans, respectively. Moreover, obesity did not reduce the efficacy of cancer immunotherapy, thus highlighting the complex interaction between obesity, the immune system, and tumor progression.

## Materials and methods

### Mice

C57BL/6J_Ola/Hsd and BALB/c female (unless stated otherwise) mice were purchased from Harlan Laboratories or bred in-house. Mice were bred and housed under specific pathogen–free conditions and maintained according to European Union regulations. Animal experiments and maintenance were approved and regulated by the Trinity College Dublin Ethics Committee and the Health Products Regulatory Authority Ireland. For obesity studies, mice were fed an HFD (60% calories from fat; Research Diets) for 6–12 wk before tumor injection, and the HFD was continued throughout the experiment. An SFD was used as a control diet. Control mice were sex- and age-matched and were from the same source as experimental mice for all experiments.

### Subcutaneous tumor models

Mice were cohoused and randomized after tumor inoculation. The B16-F10 and CT26 cell lines were purchased from the American Type Culture Collection. The MC38 cell line was kindly gifted by Arlene Sharpe (Harvard University, Cambridge, MA). B16-F10 cells (2 × 10^5^ cells/mouse) or MC38 (10^5^ cells/mouse) were injected s.c. into the right flank. Tumor growth was recorded every 2–3 d, and animals were sacrificed when they reached the tumor endpoint (tumor size ≥15 mm in diameter [D] or when tumors became ulcerous). Tumor size was calculated using the formula (D1)^2^ × (D2/2), with D1 being the smaller value of the tumor diameter. MC38 tumor-bearing mice were injected i.p. with anti–PD-1 (200 µg/mouse, clone 29F.1A12; Bio X Cell) or control IgG2A (200 µg/mouse, clone 2A3; Bio X Cell) on days 6, 9, and 12 after tumor inoculation. B16-F10 tumor-bearing mice were injected i.p. with anti–PD-1 (200 µg/mouse) or control IgG (200 µg/mouse) on days 2, 9, and 16 after tumor inoculation and injected s.c. with a cancer vaccine consisting of hs (1 h, 43°C) and irr (200 Gy) B16-F10 tumor cells (10^6^ per mouse) on days 3, 10, and 17 after tumor inoculation. Recombinant mouse IFN-γ (2.5 µg/mouse; Immunotools) was injected peritumorally on days 5, 8, 11, and 13 after tumor injection.

### Flow cytometry

For flow cytometric analysis, tumors, lungs, and dLNs were dissected, and tumors and lungs were digested with DNase I (20 U/ml; Sigma-Aldrich) and collagenase D (1 mg/ml; Roche) in RPMI 1460 for 1 h, and RBCs were lysed using ammonium chloride lysis buffer. Single-cell suspensions, prepared using a 100-µm nylon mesh, were stimulated with PMA (10 ng/ml; Sigma-Aldrich), ionomycin (500 ng/ml; Sigma-Aldrich), and brefeldin A (BFA; 5 µg/ml; Sigma-Aldrich) for 3–4 h at 37°C. Cells were stained with LIVE/DEAD Fixable Aqua Dead Cell Stain (Life Technologies) for 20 min at room temperature (RT) and fluorochrome-conjugated surface antibodies for 20 min at RT, then fixed, permeabilized, and incubated with intracellular antibodies for 30 min on ice. Data were acquired using an LSR Fortessa flow cytometer (BD Biosciences) and analyzed with FlowJo version 10 software.

### FACS antibodies

See Table 1 for a list of FACS antibodies used.

**Table 1.** FACS antibodies

Target	Clone	Supplier
**Mouse**		
CD45 BV711	30-F11	BioLegend
CD45-BV605	30-F11	BioLegend
CD3-BV650	17A2	BioLegend
CD4-AF700	RM4-5	BioLegend
CD8-APCeF780	53-6.7	eBioscience
CD19-PE/CF594	1D3	BD Biosciences
NK1.1-BV421	PK136	BioLegend
NK1.1-PerCP/Cy5.5	PK136	BioLegend
IFNγ-PerCP/Cy5.5	XMB1.2	BioLegend
GzmB-FITC	QA16A02	BioLegend
GzmB-PE/Cy7	NGZB	eBioscience
Ki-67-eF450	SolA15	eBioscience
CXCR3-APC	CXCR3-173	BioLegend
CD98-BV605	H202-141	BD Biosciences
CD98-PE	H202-141	BD Biosciences
PD-1-BV785	29F.1A12	BioLegend
PD-1-BV711	29F.1A12	BioLegend
PD-1-PE/Dazzle594	RMP1-30	BioLegend
pS6-PE	D57.2.2E	Cell Signaling Technology
pS6-PE/Cy7	D57.2.2E	Cell Signaling Technology
CD44-BV605	IM7	BioLegend
CD62L-PE/CF594	MEL-14	eBioscience
PD-L1-APC	10F.9G2	BioLegend
TIM-3-BV421	RMT3-23	BioLegend
LAG-3-eF780	C9B7W	eBioscience
TIGIT-APC	1G9	BioLegend
CD244.2-PE	m2B4 (B6)458.1	BioLegend
TCF1/TCF7-AF647	C63D9	Cell Signaling Technology
TOX-PE	REA473	Miltenyi Biotec
Eomes-eF450	Dan11mag	BioLegend
T-bet-PECF594	O4-46	BD Biosciences
TNF-BV785	MP6-XT22	BioLegend
**Human**		
CD3-AF700	UCHT1	BioLegend
CD4-APC	A161A1	BioLegend
CD8-BV605	SK1	BioLegend
CD19-APC/Cy7	HIB19	BioLegend
CD56-BV650	5.1H11	BioLegend
IFNγ-PE-CF594	B27	BD Biosciences
GzmB-FITC	GB11	BioLegend
TNF-BV421	MAb11	BioLegend
LAG-3-PE	11C3C65	BioLegend
PD-1-PE-eF610	J105	eBioscience
TIGIT-PerCP-Cy5.5	MBSA43	BioLegend
TIM-3-BV785	F38-2E2	BioLegend

### Kynurenine assay

Slc7a5 activity was measured by the flow cytometry–based kynurenine uptake assay (200 µM; Sigma-Aldrich) as previously described (Sinclair et al., 2018). Cells were stained without kynurenine (fluorescence minus one control) or with kynurenine in the presence of the selective LAT1 inhibitor BCH (25 mM; Sigma-Aldrich) as negative controls.

### I.v. staining

Mice were prewarmed in a warming chamber, and 200 µl of CD45.2-PE antibody (1.5 µg/ml, clone 104; eBioscience) was injected i.v. into the tail vein 10 min before culling. Circulating leukocytes were identified by flow cytometry by gating on CD45.2-PE–positive populations (CD45.2iv^+^), and tumor-resident leukocytes were identified by gating on CD45.2-PE–negative (CD45.2iv^−^) populations.

### Sample preparation for metabolite profiling

Mice were culled immediately by cervical dislocation. Blood was taken via cardiac puncture, collected into a microtainer tube lined with lithium heparin (Fisher Scientific), and centrifuged (10 min, maximum speed, 4°C). Plasma was subsequently snap frozen. Metabolites were extracted from plasma by adding extraction buffer, consisting of 80% methanol containing inosine-15N4, thymine-d4, and glycocholate-d4 internal standards (Cambridge Isotope Laboratories) in a 1:4 ratio of plasma to extraction buffer. Samples were then centrifuged twice (5 min, maximum speed, 4°C), and supernatants were collected.

### Metabolite analyses by mass spectrometry

Metabolite extracts (10 μl) were loaded onto a Luna-amino column (Phenomenex) using the UltiMate 3000 TPLRS LC device as previously described (Xiao et al., 2020). Subsequent analysis was performed using a Q-Exactive HF-X mass spectrometer (Thermo Fisher Scientific) as previously described (Xiao et al., 2020). Targeted processing of a subset of known metabolites was conducted using TraceFinder software version 4.1 (Thermo Fisher Scientific). Compound identities were confirmed using reference standards. In all cases, metabolite abundance was normalized using internal standards, and relative changes were assessed by comparison with metabolite extracted from the same sample type (i.e., plasma). Metabolomics data were analyzed and principal component analysis plots were generated using MetaboAnalyst 5.0 software.

### Real-time PCR

RNA was extracted from cells or tissues using TRIzol (Thermo Fisher Scientific) extraction according to the manufacturer’s instructions. Briefly, cells and tissues were resuspended in 1 ml TRIzol reagent and lysed using a tissue lyser. Cells were then mixed with 200 µl chloroform and incubated for 15 min at RT. Cells were spun down at 12,000 rpm for 15 min, and the aqueous phase was transferred to a new tube. Isopropanol was added to precipitate RNA and spun down. The RNA pellet was washed twice with 75% EtOH, and the pellet was resuspended in distilled H_2_O. cDNA was transcribed from isolated RNA using the High-Capacity cDNA Reverse Transcription Kit (Applied Biosystems) according to the manufacturer’s instructions. Real-time PCR was performed using the SYBR Select Master Mix (Applied Biosystems) according to the manufacturer’s instructions. qPCR primers were purchased from Sigma-Aldrich. To analyze relative mRNA levels, the derived values were normalized to 18s mRNA levels.

### Primer sequences in mice

See Table 2 for the primer sequences in mice.

**Table 2.** Primer sequences in mice

Gene	Primer	Sequence (5′ → 3′)
*Cxcr3*	Forward	TAC​CTT​GAG​GTT​AGT​GAA​CGT​CA
	Reverse	CGC​TCT​CGT​TTT​CC/CCA​TAA​TC
*Cxcl9*	Forward	TCC​TTT​TGG​GCA​TCA​TCT​TCC
	Reverse	TTT​GTA​GTG​GAT​CGT​GCC​TCG
*Cxcl10*	Forward	CCA​AGT​GCT​GCC​GTC​ATT​TTC
	Reverse	GGC​TCG​CAG​GGA​TGA​TTT​CAA
*18s *(housekeeping gene)	Forward	ACC​CGT​TGA​ACC​CCA​TTC​GTG​A
	Reverse	GCC​TCA​CTA​AAC​CAT​CCA​ATC​GG

### Immunohistochemistry (human endometrial cancers)

Formalin-fixed, paraffin-embedded endometrial tumor biopsies were processed for immunohistochemical staining of PD-L1 (PD-L1 concentrate, 22C3 clone; Agilent/DAKO), CD8 (CD8/144B clone; DAKO), and CD3 (LN10 clone; Leica Novocastra). PD-L1 staining was performed using the DAKO Link 48 Autostainer as per the manufacturer’s instructions. Antigen retrieval was performed using a PTLink for 20 min at 97°C in target retrieval buffer, pH 6, for PD-L1. CD8 and CD3 staining was performed using the Ventana BenchMark ULTRA system as per the manufacturer’s instructions. Antigen retrieval for CD8 was performed for 40 min at 96°C using buffer at pH 8.5. Antigen retrieval for CD3 was performed for 48 min at 96°C using buffer at pH 8.5. All antibodies were incubated at RT. Optimal dilution of CD8 was determined, using tonsil as a positive control, to be 1:40 for 32 min. Optimal dilution of CD3 was determined, using tonsil as a positive control, to be 1:400 for 32 min using buffer at pH 8.5. Optimal dilutions of PD-L1 were determined, using tonsil as a positive control tissue, to be 1:50 for 30 min. The tissue sections were then washed and incubated with HRP. The chromogen 3,3′-diaminobenzidine was used for revelation (twice for 5 min each). Tonsil was used as a positive control tissue for the IHC staining. Negative controls were run under identical conditions with diluent in place of primary antibody. The slides were counterstained with hematoxylin and rinsed in deionized water. The slides were dried in an oven (580°C) and permanently mounted.

Once dry, all the slides were scanned and digitized using the Aperio AT2 Digital Slide Scanner (Leica Biosystems) at 20× magnification, and the images were viewed, annotated, and analyzed with Aperio ImageScope 12.3 software (Leica Biosystems). The tumor areas were outlined in blue, while the IF, where present, was outlined in pink annotation. PD-L1–stained images were scored by a pathologist. The CD8- and CD3-stained images were analyzed using automated quantitative digital pathology. Macros were developed for each of the immune markers using the Aperio Nuclear version 9 Algorithm (Leica Biosystems) by training it to quantify the expression of each of the markers. Using the software tools, two annotation layers were created for each image. The number of positively stained cells was calculated for the IF and tumor areas per mm^2^ of endometrial tissue. Markup images of the analysis were generated to visualize the results.

### Human endometrial cancer cohorts

Patients with a new diagnosis of endometrial cancer were assessed in the gynecological oncology department of the Mater Misericordiae University Hospital, Dublin, Ireland. Following multidisciplinary team recommendation, patients were referred to a dedicated metabolic medicine and surgery team. All patients provided written informed consent for surgery. Ethical approval for biobanking and data collection was received from the Mater Misericordiae University Hospital under “Obesity and Uterine Cancer: Molecular Interactions and Surgical Interventions” (protocol reference 1/378/1953). A laparoscopic VSG was performed in patients deemed suitable for metabolic surgery with the goal of performing surgery within 16 wk of their referral. All women had an LNG-IUD (Mirena; Bayer) placed at diagnosis and underwent endometrial sampling every 3 mo in an outpatient setting. Endometrial samples were attained using a Pipelle (CareFusion) in an outpatient hysteroscopy clinic or under general anesthetic at the time of metabolic surgery. Patients with persistent abnormalities were scheduled for total laparoscopic hysterectomy and bilateral salpingo-oopherectomy with or without sentinel LN biopsy followed by adjuvant therapy if indicated at an appropriate interval after metabolic surgery. Patients who responded or in whom remission was achieved continued to have surveillance every 3–6 mo while the LNG-IUD remained in situ. Endometrial tissue samples were formalin fixed, paraffin embedded, sectioned, and stained with H&E. Samples were then assessed by a specialist gynecological histopathologist using the World Health Organization classification system (Emons et al., 2015).

A second cohort of endometrial cancers was selected from the University College Dublin Gynaecological Cancer Biobank for immunohistochemistry. Full informed consent was also provided under the same research protocol, “Obesity and Uterine Cancer: Molecular Interactions and Surgical Interventions” (protocol reference 1/378/1953).

### Software

Flow cytometry samples were acquired using FACSDiva and analyzed using FlowJo version 10. Real-time PCR results were acquired and analyzed using StepOne software version 2.3. Statistical analysis was performed using GraphPad Prism version 7.

### Metastatic tumor model

Mice were injected i.v. with B16-F10 tumor cells (2 × 10^5^ cells/mouse) by tail vein injection. On days 14–16 after injection, mice were sacrificed, and their lungs were dissected. The number of metastatic nodules was counted manually after bleaching in Fekete’s solution as described previously (Overwijk and Restifo, 2001). If flow cytometry was performed on the lungs, metastatic nodules were counted on fresh lungs.

### T cell isolation and stimulation

CD8 T cells were isolated from LNs or spleens using the MagniSort Mouse CD8 T Cell Enrichment Kit (Invitrogen) according to the manufacturer’s instructions. For activation assays, isolated CD8 T cells, total LN cells, or total spleen cells were cultured in anti-CD3–coated (1 µg/ml; Sigma-Aldrich) cell culture plates in the presence of IL-2 (5 ng/ml) and anti-CD28 (3 µg/ml; Sigma-Aldrich) for 48 h, unless otherwise stated.

### Seahorse metabolic flux assay

For real-time analysis of the ECAR and OCR of purified CD8 T cells, a Seahorse XFp Analyzer (Seahorse Bioscience) was used. In brief, 200,000 isolated CD8 T cells were adhered to a CellTaq (BD PharMingen)-coated 8-well XFp Cell Culture Microplate (Seahorse Bioscience). Sequential measurements of ECAR and OCR following addition of the inhibitors (Sigma-Aldrich) oligomycin (2 µM), carbonyl cyanide-*p*-trifluoromethoxyphenylhydrazone (0.5 µM), rotenone (100 nM) plus antimycin A (4 µM), and 2-deoxyglucose (30 mM) were acquired.

### TCGA analysis

TCGA uterine corpus endometrial carcinoma raw RNA-sequencing data were pseudo-aligned to the hg19 human reference genome with Kallisto (Bray et al., 2016). Clinical data from TCGA and other sources were compiled, and data were analyzed as per https://github.com/brucemoran/dyck_et_al. Median expression of IFN-γ, GzmB, and PRF1 was used to define “temperature classes.” Patient sample expression above the median were scored 1, and scores were summed. Four classes of score 0 (no genes above the median), 1, 2, and 3 (all genes above the median) were named as “cold,” “tepid,” “warm,” and “hot,” respectively. Wilcoxon rank-sum tests were conducted to determine whether the temperature classes were significantly different in their distribution of expression values of other genes of interest.

### Human peripheral blood analysis

50 ml of peripheral blood were obtained from patients with obesity attending Crumlin Hospital Dublin (three males, eight females; mean age, 42.4 [±12.62] yr; BMI, 49.4 [±6.52] kg/m^2^) or from age- and sex-matched lean healthy donors (*n* = 11) from Crumlin Hospital or Trinity College Dublin. Written informed consent was obtained from all donors. This study was approved by the Trinity College Dublin Ethics Committee (license BI-LD-310820) and the St. Vincent’s University Hospital Research Ethics Committee for blood from donors with obesity. Human PBMCs were isolated by density gradient separation using Lymphoprep (STEMCELL Technologies) according to the manufacturer’s instructions and frozen. Thawed PBMCs were either stained directly or restimulated with PMA (10 ng/ml), ionomycin (500 ng/ml), and BFA (5 µg/ml) for 3.5 h before staining for flow cytometric analysis.

### Online supplemental material

Fig. S1 shows the expression of chemokines and chemokine receptors in the tumors in SFD- and HFD-fed mice and after anti–IFN-γ treatment. Fig. S2 shows LN CD8 T cell activation and metabolic flux in lean versus obese mice. Fig. S3 shows the human CD8 T cell phenotype in patients with obesity or lean control subjects. This figure shows no overall difference in CD8 function or expression of inhibitory checkpoint receptors. Fig. S4 shows individual tumor growth curves for SFD- or HFD-fed mice with or without PVAX therapy. IFN-γ production and Ki-67 expression on CD8 TILs is measured. Metastatic nodes on the liver and lungs were evaluated. Fig. S5 shows IFN-γ, GzmB, and PRF1 genes in patients with endometrial tumors and the correlation of these genes with immune infiltration (termed tumor “temperature,” in terms of immunoscore or immune infiltration).
