# Correction: Suppressive effects of the obese tumor microenvironment on CD8 T cell infiltration and effector function

**DOI:** 10.1084/jem.2021004202072022c

**Published:** 2022-02-28

**Authors:** Lydia Dyck, Hannah Prendeville, Mathilde Raverdeau, Mieszko M. Wilk, Roisin M. Loftus, Aaron Douglas, Janet McCormack, Bruce Moran, Michael Wilkinson, Evanna L. Mills, Michael Doughty, Aurelie Fabre, Helen Heneghan, Carel LeRoux, Andrew Hogan, Edward T. Chouchani, Donal O’Shea, Donal Brennan, Lydia Lynch

Vol. 219, No. 3 | 10.1084/jem.20210042 | February 1, 2022

The authors regret that in the original version of their article, the graph in Fig. 1 E was accidentally duplicated from Fig 1 F. The corrected Fig. 1 and corresponding legend are shown here.

**Figure 1.**
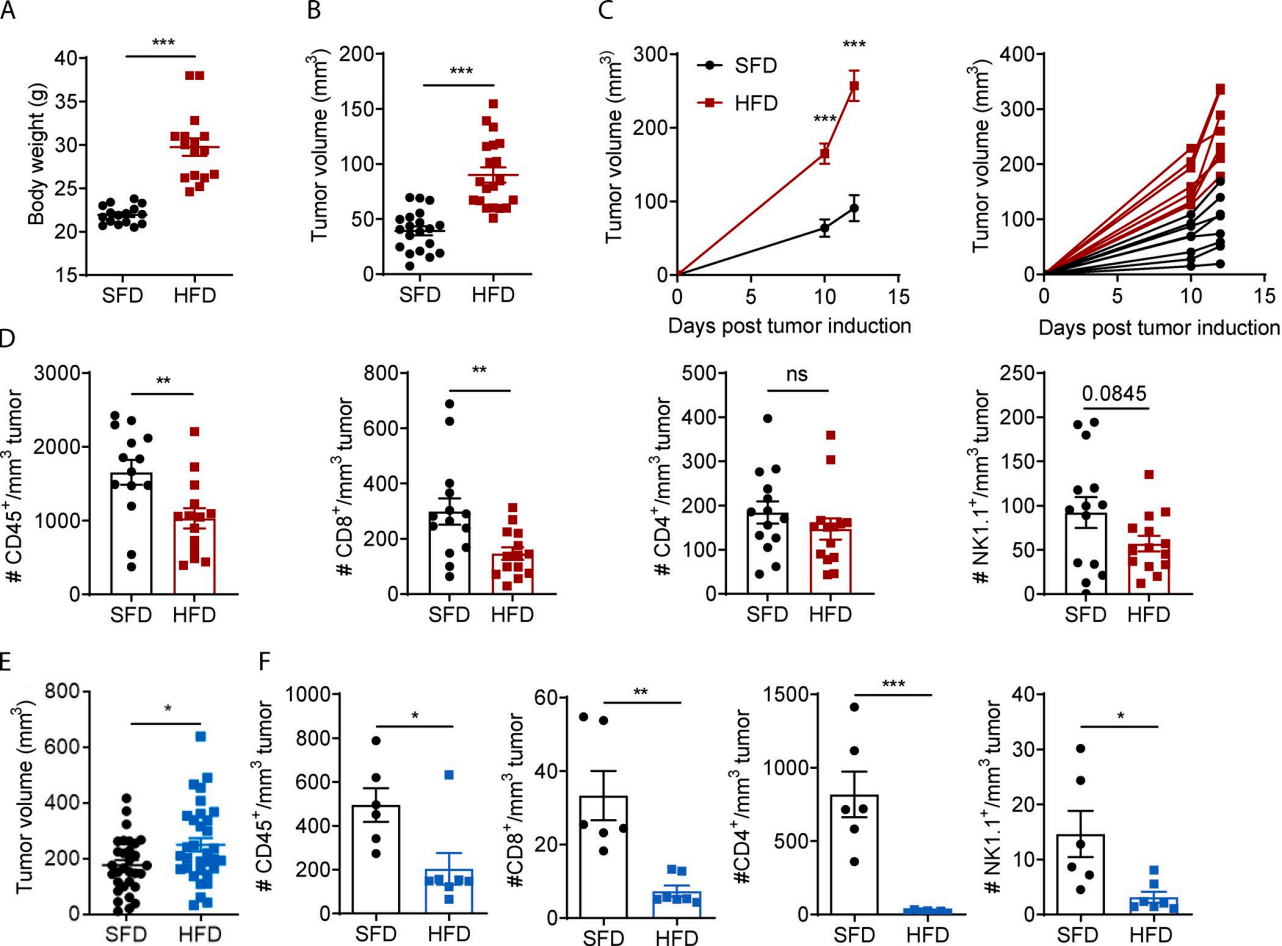
HFD-induced obesity increases tumor growth and decreases immune cell infiltration in mice. **(A–D)** C57BL/6 mice were fed an HFD (*n* = 14–16) or an SFD (*n* = 14–16) for 6–9 wk, and MC38 tumors were injected. Graphs depict weight at 9 wk for HFD (A), tumor volume on day 7 after tumor inoculation (B), and tumor growth progression (C). **(D)** Tumors from C were dissected, and immune cell infiltration was analyzed by flow cytometry. **(E)** C57BL/6 mice were fed an HFD or SFD for 10–13 wk, and mice were injected s.c. with B16-F10 tumor cells. Graph indicates tumor volume on day 11 after tumor inoculation from five pooled experiments (SFD, *n* = 31; HFD, *n* = 33). **(F)** Tumors from one experiment in E were dissected, and immune cell infiltration was analyzed by flow cytometry (SFD, *n* = 6; HFD, *n* = 7). Data are shown as individual mice (dots) and mean ± SEM. **(A, B, D, and E)** Unpaired Student’s t test. **(C)** Two-way ANOVA. *, P < 0.05; **, P < 0.01; ***, P < 0.001. This experiment was performed five times in the MC38 model and six times in the B16 model.

The error appears in PDFs downloaded before February 7, 2022.

